# Loss of PmpC in *Chlamydia trachomatis* leads to altered bacterial development and impaired recovery from IFNγ- and penicillin-induced stress

**DOI:** 10.1128/spectrum.01773-26

**Published:** 2026-10-12

**Authors:** Ailen N. Anna, María E. Panzetta, Gloria N. Bettucci Ferrero, Constanza Marín, Belkys A. Maletto, Diego D. del Balzo, Mariano Alonso Bivou, Maria T. Damiani, Robert J. Bastidas, Raphael H. Valdivia, Héctor A. Saka

**Affiliations:** 1 Centro de Investigaciones en Bioquímica Clínica e Inmunología (CIBICI), Consejo Nacional de Investigaciones Científicas y Técnicas (CONICET), Córdoba, Argentina; 2 Departamento de Bioquímica Clínica, Facultad de Ciencias Químicas, Universidad Nacional de Córdoba, Córdoba, Argentina; 3 Facultad de Ciencias Médicas, Universidad Nacional de Cuyo, Mendoza, Argentina; 4 Instituto de Medicina y Biología Experimental de Cuyo (IMBECU), Consejo Nacional de Investigaciones Científicas y Técnicas (CONICET), Mendoza, Argentina; 5 Department of Integrative Immunobiology, Duke University School of Medicine, Durham, North Carolina, USA; Institute of Parasitology, Biology Centre, ASCR, Ceske Budejovice, Czechia

**Keywords:** *Chlamydia trachomatis*, chlamydial persistence, polymorphic membrane proteins, PmpC, IFNγ-induced stress, penicillin-induced stress, *ex vivo *murine model of uterine infection

## Abstract

*Chlamydia trachomatis* is an obligate intracellular pathogen that can reversibly halt replication and developmental transitions in response to immunological or pharmacological stress, a phenomenon linked to chlamydial persistence. Although polymorphic membrane proteins (Pmps) are known adhesins, their broader roles in other pathogenesis-relevant processes remain poorly understood. Here, we characterized a *pmpC-*null mutant generated by insertional mutagenesis and found that while PmpC is not essential for primary growth, it is required for the efficient transition from reticulate bodies to infectious elementary bodies during recovery from IFNγ- and penicillin-induced stress. High-resolution microscopy and temporal expression analysis revealed that PmpC localizes to the bacterial periphery throughout the developmental cycle and is released into the lumen of pathogen-containing vacuoles (inclusions), suggesting a role beyond bacterial attachment to host cells. The absence of PmpC leads to clustered bacterial distribution and reduced bacterial mobility within the inclusion. These defects correlate with delayed cellular invasion and impaired dissemination to adjacent host cells. Notably, the significance of PmpC in penicillin-induced persistence was validated using an *ex vivo* murine uterine horn model. Altogether, our findings reveal PmpC as a multifunctional virulence factor that mediates stress recovery, regulates bacterial distribution and dynamics within the inclusion, modulates early invasion and facilitates lateral spread, expanding its recognized role as an adhesin.

**IMPORTANCE:**

*Chlamydia trachomatis* is a leading cause of sexually transmitted infections and preventable blindness globally. A significant challenge in controlling chlamydial diseases is the ability of these bacteria to enter a reversibly non-replicating “persistent” state to survive immune pressure, nutritional stress, or antibiotic treatment. In this study, we identify the surface protein PmpC as a multifunctional factor required for bacterial recovery from IFNγ- and penicillin-induced stress, in both tissue culture and an *ex vivo* murine model of uterine infection. Furthermore, we found that PmpC modulates invasion and bacterial spatial distribution within the inclusion. These findings uncover novel roles for PmpC as a virulence factor in *C. trachomatis* infection.

## INTRODUCTION

*Chlamydia trachomatis* is an obligate intracellular bacterial pathogen of significant public health impact, representing the leading global agent of bacterial sexually transmitted infections (STIs) and trachoma, the main cause of preventable infectious blindness (1–3). According to current estimates by the World Health Organization, *C. trachomatis* causes approximately 129 million new cases of STIs annually (4), and is responsible for the blindness or visual impairment of roughly 1.9 million people across 30 countries (5). *C. trachomatis* is also responsible for neonatal complications, such as ophthalmia neonatorum and pneumonia, as well as inclusion conjunctivitis in adults and a more invasive systemic infection known as lymphogranuloma venereum (6–12). A hallmark of chlamydial infection is its often asymptomatic nature, estimated to represent over 70% of *C. trachomatis* STIs in women (13). This leads to undiagnosed, chronic infections that not only sustain transmission but can also progress to severe long-term complications, such as pelvic inflammatory disease, ectopic pregnancy, and tubal factor infertility (13–18). The mechanisms by which *Chlamydia* persists within the host and evades immune clearance remain only partially understood, complicating the development of effective vaccines and targeted therapeutic strategies (19).

The biology of *Chlamydia* is defined by a complex, biphasic developmental cycle that occurs entirely within a membrane-bound vacuole termed the inclusion. This cycle involves two distinct forms: the elementary body (EB) and the reticulate body (RB) (20). The EB is the smaller, infectious form adapted for extracellular survival and pre-loaded with effectors that are secreted to facilitate entry into host cells. Upon internalization, the EB differentiates into RBs at early times post-infection. RBs are larger, metabolically active, non-infectious and specialized for active replication. Around mid-cycle, RBs begin to re-differentiate into EBs in an asynchronous process, culminating in their release via host cell lysis or extrusion, initiating new rounds of infection (20–25).

*Chlamydia* can enter a reversible non-replicative state in response to unfavorable conditions. When exposed to stressors, including IFNγ, β-lactam antibiotics, nutrient limitation, co-infection with viruses or other stress stimuli, these bacteria cease cell division and halt the generation of infectious EBs. During this persistent or stressed state, morphologically abnormal, enlarged RBs (aberrant RBs, aRBs) are frequently observed. Importantly, upon removal of the stressor, *Chlamydia* resumes cell division and complete their developmental cycle, reestablishing productive infection. This capacity for reversible developmental arrest has been postulated to contribute to the establishment of chronic, recurrent infections observed in natural disease and is a hallmark of chlamydial pathogenesis (26–37). Two stressors have been particularly well characterized in laboratory models: IFNγ and penicillin. IFNγ, primarily produced by T-lymphocytes and NK cells during host defense (38, 39), indirectly restricts chlamydial growth in epithelial cells through upregulation of indoleamine-2,3-dioxygenase, an enzyme that depletes cellular tryptophan, an essential amino acid that *C. trachomatis* cannot synthesize *de novo* (40). Under tryptophan-limiting conditions, *C. trachomatis* downregulates the production of cell-division proteins with high tryptophan content, thereby halting binary fission and triggering the transition to a persistent state (41). However, *C. trachomatis* genital serovars encode a tryptophan synthase that can generate tryptophan from indole, thereby enabling escape from IFNγ-mediated nutritional stress in environments containing indole-producing bacteria (42–46). More recent evidence indicates that IFNγ induces broader nutritional stress by depriving the pathogen of several key metabolites, including glutamate, aspartate, glycine, alanine, and glucose, as well as various TCA cycle intermediates and the ribonucleotides ATP, CTP, and UTP (47–49). This IFNγ-mediated host cell metabolic reprogramming is driven, in part, by the STAT1-dependent downregulation of the transcription factor c-Myc, which orchestrates numerous metabolic pathways exploited by *C. trachomatis*, including the TCA cycle, amino acid uptake, and nucleotide salvage (48). Penicillins are β-lactam antibiotics that block peptidoglycan cross-linking by inhibiting penicillin-binding proteins (PBPs) (50). *Chlamydia* expresses functional PBPs and produces peptidoglycan; thus, penicillin-induced persistence is most likely triggered as a stress response to impaired peptidoglycan assembly (51–54). The observation that penicillin induces recoverable persistence in both *in vitro* cultures and animal models underscores its clinical relevance, as post-treatment disease recurrence in patients has been attributed to reactivation from penicillin-induced persistent infections (51, 55–57).

The polymorphic membrane proteins (Pmps) are outer membrane-associated autotransporters conserved across *Chlamydia* species that constitute the largest protein family in the chlamydial genome. All Pmps exhibit a tripartite molecular architecture consisting of an N-terminal signal peptide, a large passenger domain (PD), and a C-terminal β-barrel translocator domain. The PD contains multiple repeats of conserved GGA(I,L,V) and FxxN tetrapeptide motifs, which serve as unique molecular signatures of this protein family (58–62). Consistent with their classification as Type V autotransporters, Pmps undergo a posttranslational proteolytic cleavage considered important for their functional maturation (23, 61, 63–69). *C. trachomatis* encodes nine distinct members (PmpA–I), which collectively account for a substantial portion (~14%) of the organism’s coding capacity, highlighting their evolutionary and biological importance (70). Proteomic analyses have confirmed that all nine *C. trachomatis* Pmps are expressed during the developmental cycle (23, 71). Although Pmps are established as oligomeric adhesins and promising vaccine candidates (60, 72, 73), their specific roles in the chlamydial stress response and recovery remain poorly understood.

In a prior genetic screen to identify factors required for recovery from IFNγ- and penicillin-induced stress (74), we identified a *C. trachomatis* mutant harboring a nonsense mutation in *pmpC*, suggesting that PmpC participates in stress-recovery mechanisms. To investigate this further, we generated a targeted *C. trachomatis pmpC*-null mutant and conducted a comprehensive phenotypic analysis in both cell culture and *ex vivo* tissue models. Our findings show that PmpC is a multifunctional virulence factor that is released from the bacterial surface into the inclusion lumen and plays important roles in IFNγ- and penicillin-induced stress recovery, bacterial spatial organization and dynamics within inclusions, host cell invasion, and dissemination to adjacent host cells.

## MATERIALS AND METHODS

### Cell culture and bacterial strains

HeLa (CCL-2, ATCC) and Vero (CCL-81, ATCC) cells were cultured in high-glucose Dulbecco’s modified Eagle’s medium (DMEM; Gibco), supplemented with 5%–10% fetal bovine serum (FBS; Natocor) without antibiotics in 5% CO_2_ at 37°C in a humidified atmosphere. Cells were passaged by trypsinization with 0.25% trypsin (Gibco) for 3 min at 37°C, followed by neutralization with complete medium. Cell stocks were preserved in cryovials using DMEM with 10% FBS and 5% DMSO at −80°C (freezer) in a Mr. Frosty cooling device (Thermo Scientific) and then transferred to −196°C (liquid nitrogen). All cell lines were routinely screened for *Mycoplasma* contamination via PCR (75). *Chlamydia trachomatis* LGV-L2 434/Bu (ATCC VR-902B) was used as the wild-type strain (L2 *wt*). The *C. trachomatis* M111 mutant was identified in a genetic screen for strains defective in recovery from IFNγ- and penicillin-induced stress (74). M111 belongs to a fully sequenced collection of mutants (76) generated via ethyl methyl sulfonate (EMS) mutagenesis as previously described (77). The *pmpC*-null mutant (L2 *pmpC::GII*) derived from L2 *wt* was generated by insertional mutagenesis using a spectinomycin resistance cassette (*aadA*) (78). To enable fluorescence-based monitoring, L2 *wt* and L2 *pmpC::GII* were transformed with the fluorescent reporter plasmids p2TK2-SW2-IncDpromoter-mCherry and p2TK2-SW2-IncDProm-RSGFP-IncDTerm (79). *Escherichia coli* DH5α was used for plasmid transformation and cloning. *E. coli* BL21 was utilized for the expression of the recombinant PmpC fragment used as immunogen for antibody generation. *C. trachomatis* stocks were stored in sucrose-phosphate-glutamate (SPG) buffer at −80°C. *E. coli* strains were maintained in Luria-Bertani (LB) broth supplemented with 20% (vol/vol) glycerol at −80°C.

### Bacterial propagation and purification

Bacteria were propagated in HeLa or Vero cell monolayers infected with elementary bodies (EBs) at specific multiplicities of infection (MOIs), followed by centrifugation at 2,000 × *g* for 30 min at 10°C. Infected cells were incubated at 37°C in 5% CO_2_ until the indicated hours post-infection (hpi) (77, 80). EBs were purified by density gradient ultracentrifugation and titered to determine inclusion-forming units per microliter (IFUs/μl) as previously detailed (23).

### *Chlamydia* transformation

Stable transformants were generated using a CaCl_2_-mediated protocol adapted for *C. trachomatis* (81). Briefly, 10^8^ inclusion-forming units (IFUs) were resuspended in 50 mM CaCl_2_ buffer (pH 7.4) and incubated with 10–20 µg of the plasmid of interest for 30 min at room temperature. This suspension was then used to inoculate confluent Vero cell monolayers in six-well plates. Selective pressure was applied at 12 hpi through the addition of spectinomycin (200 µg/mL) or penicillin (1 IU/mL), depending on the selectable marker used. Bacteria were harvested via osmotic lysis at 40–48 hpi and immediately transferred onto fresh Vero monolayers to initiate the next passage (P1). This enrichment process was repeated every 40–48 h under continuous antibiotic selection until viable transformants were observed, typically occurring within five to six passages (P5 to P6). Clonal populations were isolated using plaque purification on Vero cell monolayers as described elsewhere (77, 82). Briefly, monolayers were infected with serial dilutions of the transformant pools for 2 h in six-well plates, after which the inoculum was replaced with a semi-solid overlay. The overlay consisted of DMEM supplemented with 0.5% LE agarose (INBIO Highway), 1× DMEM (Gibco), 10% FBS (Natocor), 50 µg/mL gentamicin (Sigma-Aldrich), 1× non-essential amino acids (STEMCELL), 200 ng/mL cycloheximide, and 200 µg/mL spectinomycin (Sigma-Aldrich). Plates were incubated at 37°C in a 5% CO_2_ atmosphere for 8–14 days until plaques were visible. Individual plaques were picked using sterile tips and expanded in 96-well plates up to six-well plates, followed by hypotonic disruption and further passaging to establish stabilized, high-titer clonal stocks.

### Generation of a *pmpC* null strain

Targeted insertional inactivation of the *pmpC* gene was performed to generate the L2 *pmpC::GII* strain using the TargeTron Gene Knockout System (Sigma-Aldrich), following manufacturer’s instructions. The optimal insertion site was identified via the TargeTron target selection algorithm, focusing on high-scoring sites located near the 5′ ATG start codon. To retarget the pDFTT3aadA vector for *pmpC* disruption, specific primers were synthesized (PmpC_1_IBS ½, PmpC_1_EB S1/delta, PmpC_1_EB S2 and Universal, Table S2). pDFTT3aadA plasmid was purified using the PureYield Plasmid Midiprep System (Promega). The amplified PCR product was subsequently ligated into the BsrGI and HindIII restriction sites of the pDFTT3aadA plasmid (78). All cloning steps were performed in *E. coli* DH5α using the KCM transformation method (83). The resulting construct was verified through restriction analysis and PCR prior to *C. trachomatis* transformation. Insertion was verified by PCR using primers MOMP_F, MOMP_R, aadA_F, aadA_R, FcheckTargpmpC and RcheckTargpmpC (Table S2). Thermal cycling parameters for aadA consisted of an initial denaturation at 95°C for 10 min, followed by 35 cycles of 95°C for 30 s, 59°C for 30 s and 72°C for 2 min. For FcheckTargpmpC/RcheckTargpmpC, denaturation was followed by 35 cycles of 95°C for 30 s, 53°C for 40 s and 72°C for 5 min. For *ompA* (MOMP_F / MOMP_R), denaturation was followed by 35 cycles of 95°C for 30 s, 58°C for 15 s and 72°C for 30 s.

### IFNγ and penicillin stress induction and recovery assays

IFNγ- and penicillin-induced stress assays were carried out as described previously (74). Briefly, HeLa cells were pre-treated with IFNγ (15 ng/mL) for 24 h before infection (MOI 0.8) in the continued presence of the cytokine. At 24 hpi, recovery (IFN-Rec) was initiated by washing cells with PBS and replacing the medium with IFNγ-free DMEM supplemented with 10% FBS and an excess of tryptophan (100 mg/L) for 24 h. For penicillin-induced stress, HeLa cells were infected (MOI 0.8) with the addition of 1 IU/mL penicillin G at the time of infection. At 24 hpi, cells were washed with PBS and penicillin-free DMEM supplemented with 10% FBS was added to allow recovery for 24 h (PEN-Rec). Untreated controls (UTs) consisting of HeLa cells infected in the absence of stressors, as well as non-recovery controls constantly treated with stressors throughout the experiment (IFN-NoRec or PEN-NoRec), were included. For infectious progeny quantification, infected cells were lysed with distilled water and 5× SPG buffer was added to reach a 1× final concentration. These lysates were then serially diluted and used to infect fresh HeLa monolayers for 30 h. Inclusions were fixed with 4% para-formaldehyde (PFA), stained with rabbit anti-CT043 followed by Alexa Fluor 488-conjugated anti-rabbit secondary antibodies, and quantified using an ImageJ macro developed in our laboratory to determine the IFUs/μl. At all times, progeny was normalized to the input. To calculate the input for each strain, infected HeLa cells (MOI 0.8) were PFA-fixed at 30 hpi, and inclusions were stained and quantified as described above.

### Quantitative PCR (qPCR) for determination of genome copy numbers

Quantification of genome copy numbers was carried out, as described previously (74). Briefly, total DNA was extracted from HeLa cells infected with either L2 *wt* or L2 *pmpC::GII* strains following recovery from IFNγ- or penicillin-induced stress utilizing the DNeasy kit (Qiagen) according to the manufacturer’s instructions. Genome copies were quantified via SYBR Green-based qPCR targeting the *C. trachomatis ompA* gene using a 7500 Real-Time PCR system (Applied Biosystems). For the construction of a standard curve, we employed the previously described pET24d_OmpA plasmid (74), which was purified utilizing the PureYield Plasmid Miniprep System (Promega) and quantified via Qubit fluorometry (Thermo Fisher Scientific) to establish the standard curve. Amplification was performed using primers MOMP_F and MOMP_R (Table S2). Thermal cycling parameters consisted of an initial denaturation at 95°C for 10 min, followed by 40 cycles of 95°C for 15 s and 60°C for 1 min.

### RNA extraction and reverse transcription

For the transcriptional profiling of the *pmpABC* operon, total RNA was harvested from HeLa cells infected with either L2 *wt* or L2 *pmpC::GII* strains at 44 hpi utilizing TRIzol reagent (Invitrogen). RNA yield and purity were evaluated by measuring absorbance at 260 nm with a NanoDrop Lite spectrophotometer (Thermo Scientific). To ensure the removal of residual genomic DNA, 3 μg of total RNA was subjected to DNase treatment. Subsequently, 1 μg of the DNA-free RNA was reverse-transcribed into cDNA employing LunaScript RT SuperMix (New England Biolabs). The resulting cDNA served as the template for PCR analysis using specific primers detailed in Table S2 (aadA_F, aadA_R, FcheckTargpmpC, RcheckTargpmpC, pmpAgene_F, pmpAgene_R, pmpBgene_F, pmpBgene_R, pmpCgene_F, pmpCgene_R). Thermal cycling parameters for *aadA* and FcheckTargpmpC/RcheckTargpmpC were the same as described above. For the *pmpABC* operon, denaturation at 95°C for 10 min was followed by 35 cycles of 95°C for 30 s, 50°C for 15 s, and 72°C for 30 s.

### Transmission electron microscopy

For ultrastructural analysis, HeLa cells infected with *C. trachomatis* L2 *wt* or L2 *pmpC::GII* at the specified conditions were detached with 0.5% trypsin-EDTA solution, collected by centrifugation at 1,500 × *g* for 5 min and fixed with 2.5% glutaraldehyde in 0.1 M sodium cacodylate buffer. Cell pellets were subsequently processed for post-fixation, dehydration, embedding, and ultramicrotomy as described (84). Ultrathin sections were examined at the Electron Microscopy Center, Facultad de Ciencias Médicas, Universidad Nacional de Córdoba. To quantify chlamydial developmental forms, at least 15 inclusions per condition were randomly selected for the enumeration of EBs and RBs. Digital images were prepared using Adobe Photoshop CS6 (Adobe Systems Inc.).

### Immunofluorescence microscopy and image analysis

Indirect immunofluorescence assays were performed on cell monolayers fixed with 4% paraformaldehyde (PFA) for 10 min, permeabilized with 0.1% Triton X-100 in PBS, and blocked with 5% bovine serum albumin (BSA). Primary antibodies used were: rabbit anti-Slc1 (CT043) (23), mouse anti-MOMP (Abcam, ab20881), and a mouse polyclonal antibody raised against a *C. trachomatis* L2 PmpC-derived recombinant peptide (generated in this study, as detailed below). Rabbit anti-CT043 and mouse anti-MOMP antibodies were conveniently used to label *C. trachomatis* cells. Samples were incubated with primary antibodies for 1 h at room temperature (anti-CT043 and anti-MOMP) or overnight at 4°C (anti-PmpC), followed by detection with Alexa Fluor 488- or 594-conjugated anti-mouse or anti-rabbit secondary antibodies. Host and bacterial DNA were stained with DAPI (4′,6-diamidino-2-phenylindole, ThermoFisher Scientific). Coverslips were mounted using Mowiol 4-88 (Sigma-Aldrich). For animal tissue samples, autofluorescence was quenched with 0.1% Sudan Black B in 70% ethanol, followed by permeabilization with 0.25% Triton X-100 and blocking with 5% BSA. Primary antibodies were incubated overnight at 4°C and detected with appropriate secondary antibodies. Standard fluorescence and wide-field images were acquired using a Leica DMI8 epifluorescence microscope or a Cytiva InCell Analyzer 2500HS high-content imaging system. Confocal microscopy was performed on either an Olympus FV1200 or a Zeiss LSM 980 confocal microscope. For high-resolution spatial analysis, super-resolution microscopy was conducted using a Zeiss LSM 980 Airyscan II confocal microscope equipped with a Plan-Apochromat 63× objective. Image processing and quantitative analyses were performed using ImageJ (win64) and Adobe Photoshop CS6 (Adobe Systems Inc.). Co-localization was assessed by drawing straight-line regions of interest (ROIs) across chlamydial inclusions. Pearson’s correlation coefficients (R) and multichannel plot profiles were calculated along these ROIs to analyze the spatial relationship between the PmpC signal and mCherry-expressing bacteria. For quantitative fluorescence analysis, individual chlamydial inclusions were delineated as ROIs on red channel images. A fixed intensity threshold was applied to each ROI to segment mCherry-positive bacteria, followed by the measurement of fluorescence area and integrated intensity (arbitrary units). For time-lapse microscopy, HeLa cells were either co-infected with L2 *wt* GFP and L2 *pmpC::GII* mCherry (MOI: one each) or singly infected with mCherry-expressing strains (MOI: 1). Super-resolution time-lapse image stacks were acquired at 44 hpi (200 frames at 0.26-s intervals) using the Zeiss LSM 980 Airyscan II confocal microscope. For particle tracking, mCherry-positive bacteria within inclusions were automatically detected and tracked using the TrackMate plugin in ImageJ (85). Detection was performed using a Laplacian of Gaussian (LoG) detector with an estimated spot diameter of 0.8 μm. Resulting trajectories were used to calculate total distance traveled and average velocity.

### Adhesion and invasion assays

To evaluate bacterial adherence and internalization, we employed a differential staining technique. HeLa monolayers were infected with mCherry-expressing L2 *wt* or L2 *pmpC::GII* (MOI: 10) for 1 or 6 h. Following fixation with PFA 4%, differential immunofluorescence staining was performed without cell permeabilization to distinguish between extracellular and intracellular bacteria. Extracellular bacteria were detected using a mouse anti-MOMP primary antibody (Abcam, ab20881) followed by an Alexa Fluor 488-conjugated secondary antibody. Under these conditions, extracellular bacteria appeared yellow (mCherry+/Alexa Fluor 488+), while internalized bacteria remained exclusively red (mCherry+/Alexa Fluor 488-). DNA was stained with DAPI (ThermoFisher Scientific). The total number of adherent bacteria per cell was determined by quantifying all mCherry-positive particles. The invasion rate, representing the proportion of internalized bacteria, was calculated by subtracting the number of extracellular bacteria (green) from the total number of bacteria (red) and dividing the difference by the total number of bacteria (red). Samples were visualized using a Zeiss LSM 980-Airyscan II confocal microscope and images were processed using Adobe Photoshop CS6 (Adobe Systems Inc.).

### Plaque and dissemination assays

To assess lateral dissemination, plaque assays were performed on HeLa monolayers infected with mCherry-expressing L2 *wt* and L2 *pmpC::GII* strains, as previously detailed (77), with the exception that infections were carried out at an MOI of 0.01. At 2 hpi, the infectious inoculum was removed and replaced with a semi-solid overlay medium consisting of DMEM supplemented with 0.5% LE agarose (INBIO Highway), 10% FBS (Natocor), 50 µg/mL gentamicin (Sigma-Aldrich), 1× non-essential amino acids (STEMCELL), and 200 ng/mL cycloheximide (Sigma-Aldrich). Infected plates were incubated at 37°C in 5% CO_2_ for 15–20 days to allow for the expansion of lysis plaques. Live-cell fluorescence images were acquired at 4× magnification using a Cytiva InCell Analyzer 2500HS system. The total plaque area per field (arbitrary units) was quantified for both strains using ImageJ to determine and compare their respective dissemination capacities.

### Detection of extracellularly released PmpC

To assess the release of PmpC into the extracellular environment, HeLa monolayers in six-well plates were infected with the L2 *wt* strain and harvested at 18, 24, and 48 hpi. A crude subcellular fractionation was achieved by subjecting infected monolayers to hypotonic shock with 400 μL of sterile water per well for 15 min at 37°C. The resulting lysates were centrifuged at 15,000 × *g* for 15 min at 4°C to partition the bacterial pellet from the host cell supernatant. The pellet fraction was resuspended in lysis buffer comprising 20 mM Tris-HCl (pH 8), 5 mM EDTA, 50 mM NaCl, 1% Triton X-100, 1 mM PMSF, protease inhibitor cocktail (Roche), 1% SDS, 15 mM DTT, and 14.3 mM 2-mercaptoethanol. Both the pellet and supernatant fractions were homogenized in 2× Laemmli sample buffer, sheared by sonication on ice (20% amplitude for 1 min), and sequentially incubated at 65°C for 10 min and 100°C for 10 min to ensure complete protein denaturation. Protein distribution was resolved by 10% SDS-PAGE and analyzed by immunoblotting using specific antibodies against PmpC (generated in this study, detailed below) and MOMP (Abcam, ab20881).

### Western blot analysis

At the end of the experiment, cells were washed with PBS and collected by scraping. Protein extracts were obtained by lysing cells in pre-heated Laemmli sample buffer according to manufacturer protocols (Bio-Rad). Lysates were sheared via sonication, heated at 100°C for 5–10 min to ensure denaturation, and clarified by centrifugation. Equivalent protein loads were resolved by 10% SDS-PAGE and electroblotted onto 0.45-μm nitrocellulose membranes (Bio-Rad) utilizing a Trans-Blot SD Semi-Dry Transfer Cell at 20 V for 30 min. Membranes were blocked with 5% non-fat dry milk in Tris-buffered saline containing 0.2% Tween 20 (TBS-T) for 1 h. Following blocking, membranes were probed with primary antibodies overnight at 4°C under constant agitation. After sequential washes with TBS-T and TBS, IRDye-conjugated secondary antibodies (LI-COR Biosciences) were applied for 1 h at room temperature. Immunoreactive bands were visualized and captured using an Odyssey CLx infrared imaging system (LI-COR Biosciences).

### *Ex vivo* murine uterine model

Uterine horns were harvested from 8- to 12-week-old female C57BL/6 mice. Following euthanasia by CO_2_ inhalation and subsequent cervical dislocation, the reproductive tracts were aseptically extracted and immediately submerged in DMEM supplemented with 10% FBS and 20 µg/mL gentamicin. To prepare the tissue explants, the cervix and vagina were removed via scalpel excision. Each uterine horn was subsequently partitioned into two segments of approximately equal length (3–4 mm each) by transverse incision, resulting in four standardized sections per animal. Individual tissue segments were transferred to 24-well plates containing complete DMEM (10% FBS, 20 µg/mL gentamicin) and maintained in a humidified incubator at 37°C with 5% CO_2_ until infection. All explants were infected within 30 min of sectioning. Each tissue segment was inoculated with 2 × 10^7^ IFUs of L2 *wt* or L2 *pmpC::GII* strains. For untreated conditions, the infection proceeded until 72 hpi, with PBS washes and medium replacement performed every 24 h. For penicillin-induced stress, tissues were infected as described above; however, at 24 hpi, each segment was washed with PBS and the medium was supplanted with fresh medium containing 0.5 IU/mL penicillin G. This condition was maintained for 24 h. At 48 hpi, the tissues were again washed with PBS, and the antibiotic-containing medium was replaced with antibiotic-free complete medium to allow for recovery for an additional 24 h until the 72 hpi endpoint. Then, samples were collected for infectious progeny quantification, indirect immunofluorescence, and histological analysis.

### Quantification of infectious progeny from tissue homogenates

Infected tissue sections and non-infected controls were washed with PBS and transferred to 1.5-mL Eppendorf tubes containing 1 mL of 1× SPG buffer. Tissues were homogenized using an ULTRA-TURRAX T25 disperser (IKA-Labortechnik) for three 10–15 s pulses at 9,500 rpm. To remove large cellular debris, homogenates were centrifuged at 500 × *g* for 5 min at 4°C. The resulting supernatants were collected and sonicated on ice (40% amplitude; three 15-s series with 5 s on/off intervals). Finally, the supernatants were utilized to infect HeLa cell monolayers to determine the IFU counts (23).

### Histological analysis and H&E staining

Infected *ex vivo* cultures were fixed in 10% neutral buffered formalin (pH 7; Biopack). Tissues were subsequently dehydrated through a graded series of ethanol, cleared in xylene (Cicarelli), and embedded in liquid paraffin at 60°C. Sections of 4 μm were cut using a manual rotary microtome (Thermo Scientific HM 325), resolved in a 45°C water bath to eliminate folds, and mounted onto glass slides. For both hematoxylin and eosin (H&E) staining and subsequent immunofluorescence processing, sections were first deparaffinized in xylene and rehydrated through a descending alcohol series, followed by distilled water. For H&E, sections were stained with activated hematoxylin (Biopur) for 40 s. Color bluing was achieved by rinsing in water for 3 min. The sections were then counterstained with eosin (Biopack) for 10 min, dehydrated through an ascending alcohol series, and finally mounted using Canada Balsam (Biopack) and coverslips.

### Generation of recombinant PmpC and polyclonal antibody production

A recombinant fragment of the *C. trachomatis* L2 434/Bu PmpC passenger domain corresponding to amino acids 596–977 (nucleotides 1,786–2,931), was amplified by PCR using primers FHisp-PmpC2 and RHisp-PmpC2 (Table S2). Thermal cycling conditions consisted of an initial denaturation at 95°C for 10 min, followed by 35 cycles of 95°C for 30 s, 55°C for 15 s and 72°C for 45 s. The PCR product was cloned into the pHis-Parallel expression vector to incorporate an N-terminal 6xHis-tag. Cloning steps were performed in *E. coli* DH5α and the resulting construct verified by NcoI/StuI restriction digestion. For protein production, the construct was transformed into *E. coli* BL21(DE3) pLysS and expression was induced with 0.5–2 mM IPTG for 2–5 h at 37°C. Solubility assays (QIAexpressionist, Qiagen) localized the recombinant PmpC fragment to the insoluble inclusion body fraction. The protein was subsequently solubilized under denaturing conditions (8 M urea) and purified via Ni-NTA affinity chromatography (Qiagen), according to the manufacturer’s instructions. To generate polyclonal antibodies, 8- to 12-week-old female C57BL/6 mice were immunized with three 60-µg doses of the denatured recombinant protein on days 0, 15, and 30. Prior to each immunization, the protein was formulated with the CpG-ODN/Coa-ASC16 adjuvant system, as previously described (86). This liquid crystal-based system consisted of CpG-ODN 1826 (Operon Technologies), containing a full phosphorothioate backbone and prepared in sterile, pyrogen-free 0.9% NaCl, and ASC-16 (Sigma-Aldrich). The resulting polyclonal antiserum was validated for specificity in immunofluorescence and immunoblot assays.

### Statistics

Statistical analyses were performed using GraphPad Prism (version 9.2.0). Data are presented as means ± standard deviation (SD) from at least three independent experiments, unless otherwise specified. Normality was assessed using the Shapiro-Wilk or Kolmogorov-Smirnov test to determine the appropriate statistical approach. Comparisons between two groups (L2 *wt* versus L2 *pmpC::GII* within a specific condition) were performed using an unpaired Student’s *t*-test (for normally distributed data), the Mann–Whitney *U* test (for non-parametric data), or χ² test (for comparison of proportions), as specified in the corresponding figure legends. In all cases, a *P*-value < 0.05 was considered statistically significant (*P < 0.05; **P < 0.01; ***P < 0.001; ****P < 0.0001; ns, not significant).

## RESULTS

### PmpC is required for *C. trachomatis* recovery from IFNγ- and penicillin-induced stress

To identify chlamydial factors involved in recovery from IFNγ- and penicillin-induced stress, we previously conducted a forward genetic screen using a fully sequenced *C. trachomatis* L2 chemical mutant library (74). While that study established the role of the secreted protein Ptr/CTL0175 in the efficient recovery from IFNγ-induced stress, several additional mutants exhibited a reduced ability to generate infectious progeny upon stress removal. For instance, the mutant M111 consistently displayed a marked reduction in IFU generation upon recovery from both IFNγ- and penicillin-induced stress (Fig. S1). Whole-genome sequencing of M111 revealed several non-synonymous single nucleotide variants (SNVs). Notably, we identified a C3061T substitution in the *pmpC* gene (CTL0671) that introduces a premature stop codon (N1020*) (Table S1). To test whether the loss of PmpC was responsible for the observed defects in M111, we generated a clean insertional mutant of *pmpC* by introducing a group II intron carrying a spectinomycin resistance cassette (*aadA*-GII) (78) into the *pmpC* coding sequence. PCR analysis of the resulting L2 *pmpC::GII* strain confirmed the site-specific insertion of the *aadA* cassette into the *pmpC* gene (Fig. S2). Because *pmpC* is part of the *pmpABC* operon, we next evaluated the transcriptional integrity of the locus. RT-PCR analysis demonstrated that while transcription of the upstream *pmpA* and *pmpB* genes was preserved in the L2 *pmpC::GII* mutant, the *pmpC* mRNA was larger, consistent with the inclusion of the ~2.3-kb *aadA* cassette (Fig. S3). This confirms that the operon is actively transcribed and the *pmpC* promoter remains functional.

**Fig 1:**
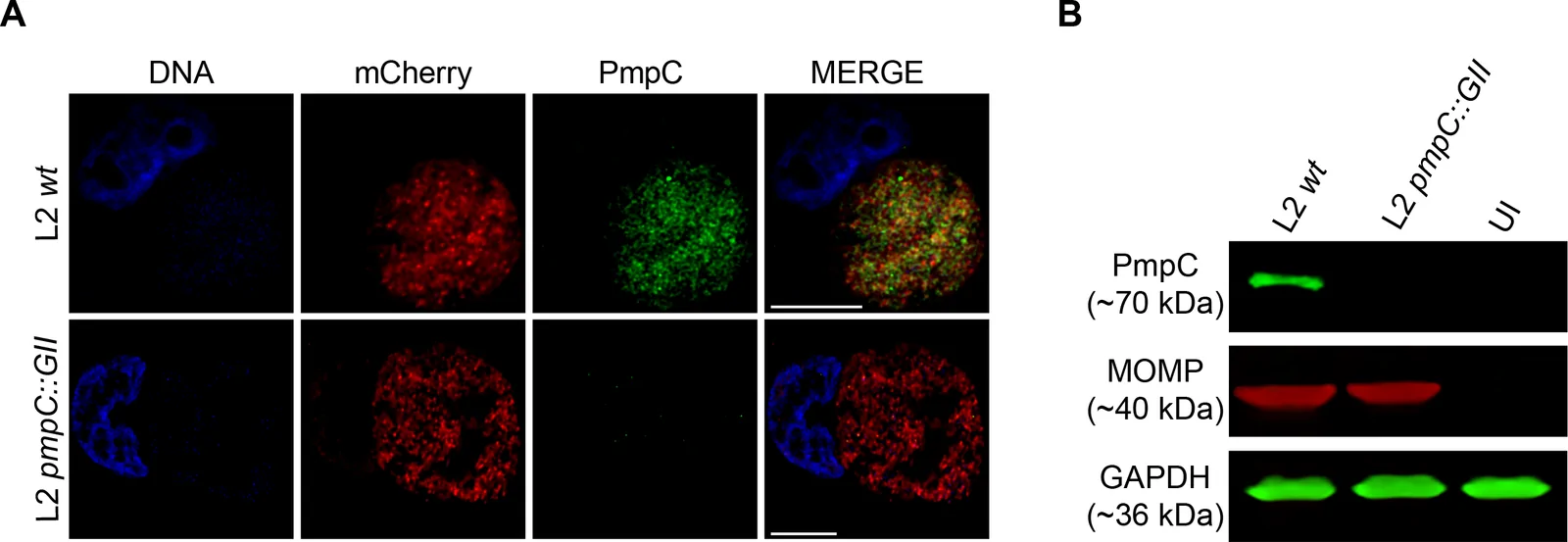
Insertional disruption of *pmpC* results in lack of PmpC expression. (**A**) PmpC expression is observed in L2 *wt* but not in L2 *pmpC::GII* inclusions. Representative confocal images of HeLa cells infected with *C. trachomatis* L2 *wt* or L2 *pmpC::GII* at 30 hours post infection (hpi). *C. trachomatis* strains express mCherry (red), DNA is stained with DAPI (blue), and cells were immunoassayed with anti-PmpC (green). Scale bars represent 10 µm. (**B**) Immunoblot with anti-PmpC confirms lack of PmpC expression in L2 *pmpC::GII*. Total protein extracts were obtained from HeLa cells at 30 hpi. Anti-MOMP and anti-GAPDH antibodies were used as loading controls. UI: uninfected. kDa: kilodaltons.

We next confirmed that the *pmpC::GII* insertion abolished PmpC protein expression using a mouse polyclonal antibody raised against a recombinant fragment of the PmpC passenger domain (aa 596–977). Immunofluorescence microscopy of HeLa cells infected with the L2 *wt* strain showed PmpC expression within the inclusions, which was absent from cells infected with L2 *pmpC::GII* (Fig. 1A). This loss of PmpC expression was further confirmed by immunoblot analysis of infected cell lysates, which indicated the presence of a ~70-kDa PmpC band in L2 *wt* infections that was completely absent in L2 *pmpC::GII* infections (Fig. 1B). Together, these results confirm the successful knockout of PmpC in the L2 *pmpC::GII* strain.

**Fig 2:**
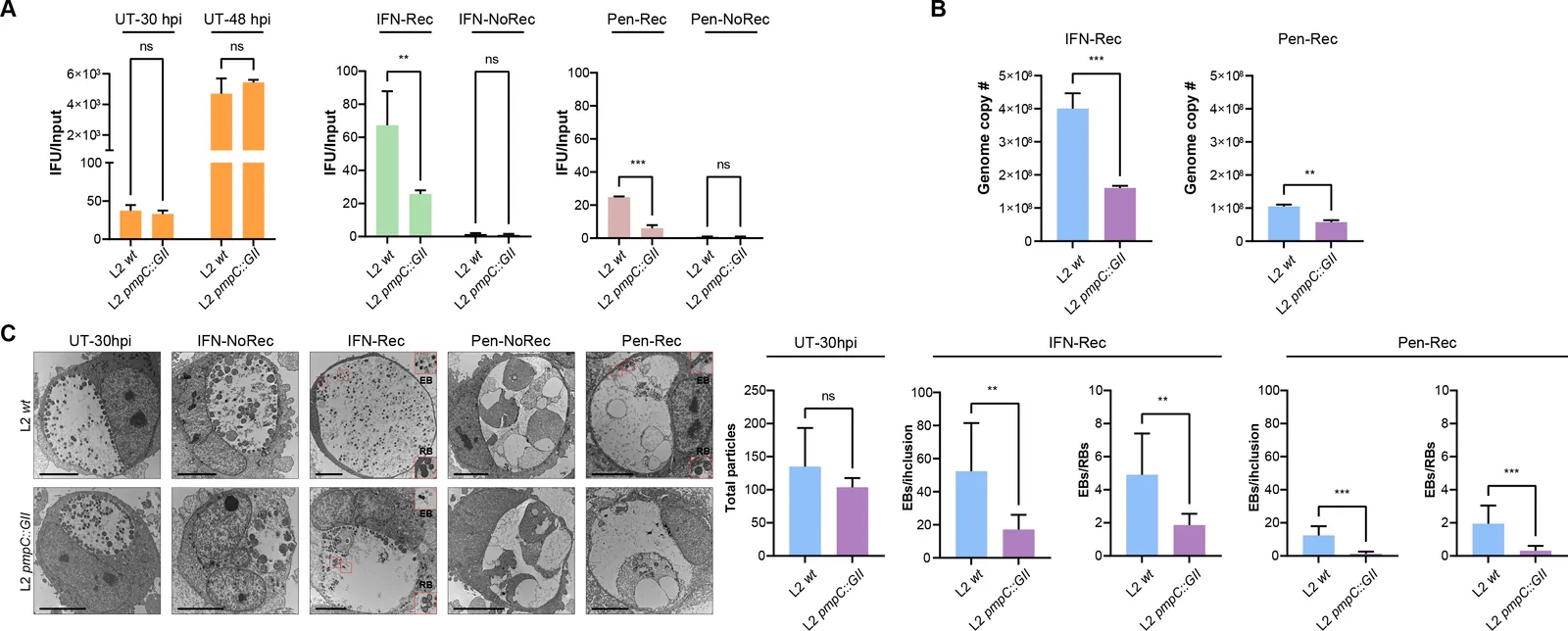
PmpC facilitates recovery of *C. trachomatis* from IFNγ- and penicillin-induced stress. (**A**) *C. trachomatis* L2 *wt* and L2 *pmpC::GII* produce infectious progeny at similar levels in the untreated condition at 30 and 48 hpi (left), while L2 *pmpC::GII* exhibits impaired production of infectious progeny post IFNγ- (middle) and penicillin- (right) induced stress. IFU/Input: infectious forming units normalized per input. UT: untreated (30 hpi). IFN-Rec: recovery post IFNγ removal (24 h recovery, 48 hpi total). IFN-NoRec: continuous presence of IFNγ (48 hpi). Pen-Rec: recovery post penicillin removal (24 h recovery, 48 hpi total). Pen-NoRec: continuous presence of penicillin (48 hpi). (**B**) L2 *pmpC::GII* shows decreased genome accumulation upon recovery from IFNγ- and penicillin-induced stress. Genome copy number was quantified by real-time PCR during recovery for both stressors using *ompA*-specific primers. (**C**) Left: lack of PmpC impairs RB to EB transition upon recovery from IFNγ- and penicillin-induced stress. Representative TEM images of the UT, IFN-NoRec, IFN-Rec, Pen-Rec, and Pen-NoRec conditions of HeLa cells infected with L2 *wt* or L2 *pmpC::GII*. Red squares indicate regions of interest highlighting CT particles displaying typical ultrastructural features of EBs and RBs. Scale bars: 10 µm. Right: quantification of total chlamydial particles (UT condition), EBs/inclusion, and EB/RB ratio (IFN-Rec and Pen-Rec conditions) as visualized by TEM. At least 15 inclusions per condition were analyzed (right). (**A–C**) Data are expressed as the mean ± SD of three replicates. Asterisks indicate statistically significant differences as determined by Student’s *t*-test (**P < 0.01, ***P < 0.001, ns: not significant).

We next induced cellular stress in infected cells with IFNγ and penicillin treatment to determine whether we could recapitulate the recovery defects observed in the M111 mutant. In untreated cells, infection with L2 *pmpC::GII* yielded similar levels of infectious EBs as L2 *wt* at both 30 and 48 hpi (Fig. 2A). Both strains were restricted by IFNγ or penicillin, but upon the removal of the stressors, L2 *pmpC::GII* failed to recover efficiently with a marked decrease in the production of infectious EBs (Fig. 2A). To investigate the mechanism underlying the stress recovery defects, we first assessed bacterial genome replication during the recovery phase by quantitative PCR. The number of L2 *pmpC::GII* genomes was significantly reduced compared with L2 *wt* during recovery from either IFNγ or penicillin stress (Fig. 2B). This result indicates that loss of PmpC impairs the ability of *C. trachomatis* to resume genome replication following stress removal. We examined the developmental forms present during stress recovery by TEM and determined that under untreated conditions, both strains showed similar total numbers of chlamydial particles per inclusion (Fig. 2C). However, during recovery from both stressors, L2 *pmpC::GII* inclusions displayed significantly fewer EBs per inclusion and a reduced EB/RB ratio. Collectively, these results show that PmpC is dispensable for *C. trachomatis* growth in the absence of stressors but required for recovery from IFNγ- and penicillin-induced stress. Our data indicate that this impaired recovery is characterized by reduced genome replication efficiency and altered developmental progression from RBs to infectious EBs.

### PmpC localizes to the bacterial surface, is released into the inclusion lumen, and maintains expression during stress and recovery

**Fig 3:**
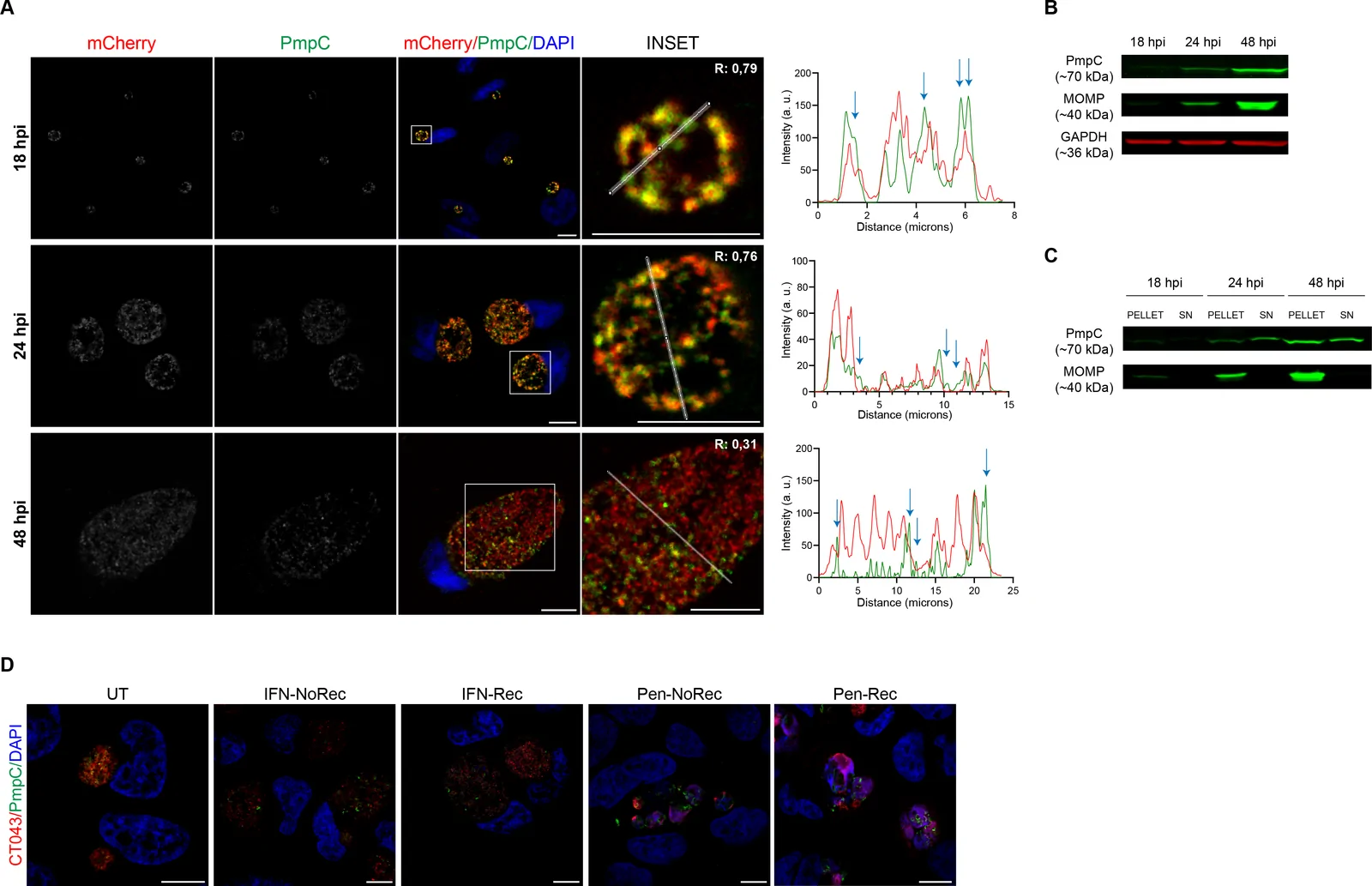
PmpC is expressed at mid to late stages of *C. trachomatis* developmental cycle, released to the lumen of the chlamydial inclusion and maintains expression during stress and recovery. (**A**) Immunofluorescence microscopy analysis of PmpC expression at 18, 24 and 48 hpi exhibits partial colocalization with bacterial cells. Left: representative confocal images of HeLa cells infected with L2 *wt* at the indicated hpi constitutively expressing mCherry (red). DNA was stained with DAPI (blue). White squares indicate regions of interest that are shown at higher magnification (INSET). Pearson’s correlation coefficients (R) for the insets are provided to assess the degree of colocalization. Scale bars represent 10 µm. Right: representative multichannel line-scan profiles corresponding to the white lines in the insets indicate PmpC signal within the inclusion outside of bacterial cells. Plots depict fluorescence intensities in arbitrary units (a.u.) for PmpC (green) and mCherry-expressing bacteria (red). Blue arrows highlight PmpC signal within the inclusion that does not overlap with bacterial cells. (**B**) Total PmpC protein levels increase as the developmental cycle progresses. Immunoblot analysis of total protein extracts from infected HeLa cells confirms expression of PmpC at 18 to 48 hpi. Mouse anti-MOMP (MOMP) and rabbit anti-GAPDH (GAPDH) served as chlamydial and host cell loading controls, respectively. kDa: kilodaltons. (**C**) Fractionation of infected HeLa cells at the indicated hpi reveals PmpC in both the bacterial pellet (PELLET) and the soluble supernatant (SN) fractions. The absence of MOMP in the SN fraction indicates integrity of the bacterial cells during fractionation. Fractions were immunoblotted with anti-PmpC (PmpC) and anti-MOMP (MOMP). kDa: kilodaltons. (**D**) PmpC is expressed during stress and recovery conditions. Representative confocal images of HeLa cells infected with L2 *wt* under untreated (UT), stress (IFN-NR, Pen-NR) and stress recovery (IFN-Rec, Pen-Rec) conditions. UT: untreated (24 hpi), IFN-NoRec: continuous presence of IFNγ (48 hpi), IFN-Rec: recovery post IFNγ removal (24 h recovery, 48 hpi total), Pen-NoRec: continuous presence of penicillin (48 hpi), Pen-Rec: recovery post penicillin removal (24 h recovery, 48 hpi total). Cells were co-stained with anti-CT043 for bacterial labeling (red), anti-PmpC (green), and DAPI (blue, DNA). Scale bars represent 10 µm.

Upon establishing that PmpC is an important factor in stress recovery, we sought to characterize its expression and localization during standard *C. trachomatis* development. We first examined PmpC expression throughout the chlamydial developmental cycle at 18, 24, and 48 hpi using immunofluorescence microscopy. PmpC signal was detectable at 18 hpi, becoming increasingly evident at 24 and 48 hpi (Fig. 3A). This temporal pattern was further corroborated by immunoblotting of infected HeLa cell lysates, which showed accumulation of PmpC as the infection progressed from 18 to 48 hpi, mirroring the increase in total chlamydial load (Fig. 3B). Interestingly, our microscopy data showed that PmpC localization only partially overlapped with mCherry-expressing bacteria. While the association was high at earlier time points, it became less pronounced by the end of the developmental cycle, with Pearson coefficients dropping from 0.79 and 0.76 at 18 and 24 hpi, respectively, to 0.31 at 48 hpi (Fig. 3A). In agreement with this, line-scan analysis of fluorescence intensity across individual inclusions revealed a distinct PmpC signal that did not overlap with mCherry-labeled bacteria (Fig. 3A). This observation suggested that a fraction of PmpC might be released into the inclusion lumen. To test this hypothesis, we performed fractionation of infected cells using hypotonic shock followed by centrifugation to partition the samples into a particulate fraction containing the chlamydial organisms (PELLET), and a soluble fraction representing the inclusion lumen contents and host cytosol (SN). PmpC was detected in both the bacterial pellet and supernatant fractions at 18, 24, and 48 hpi, with increasing amounts over time (Fig. 3C). Importantly, the non-secreted major outer membrane protein (MOMP) remained restricted to the pellet at all time points. The detection of PmpC in the supernatant fraction in the absence of MOMP leakage further supports the specific release of PmpC from bacterial cells during infection. This release suggests that PmpC may exert functions within the inclusion lumen.

We next investigated whether PmpC expression is maintained under stress conditions and during recovery. Immunofluorescence microscopy revealed that PmpC is expressed in the presence of IFNγ- and penicillin-induced stress and upon removal of the stressors (Fig. 3D). Interestingly, in penicillin-treated cultures, PmpC was observed surrounding aberrant RB structures, a localization pattern resembling that previously described for PmpD and PmpI (87).

**Fig 4:**
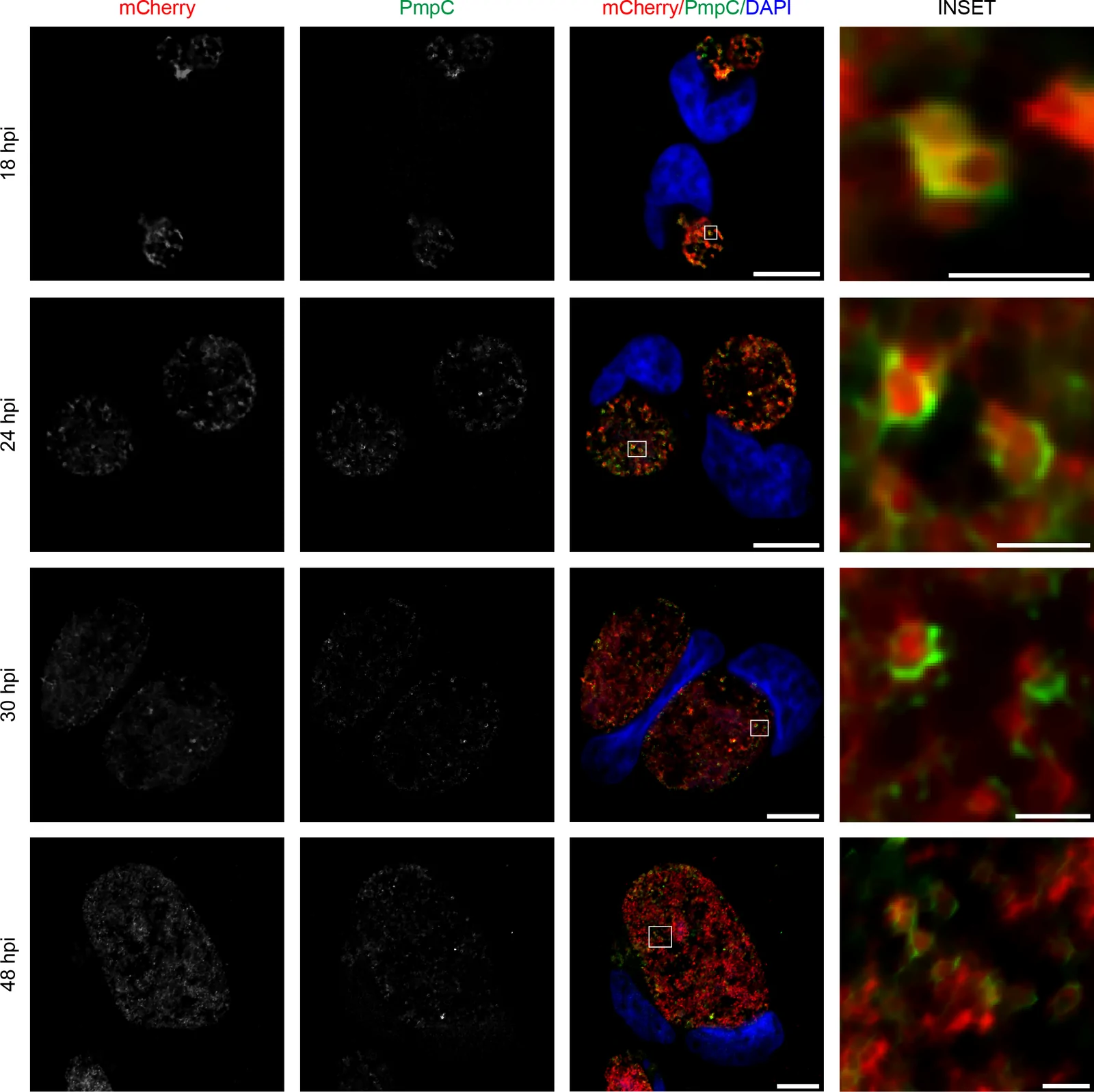
PmpC localizes to the surface of *C. trachomatis*. Representative super-resolution confocal images of HeLa cells infected with L2 *wt* at the indicated hpi constitutively expressing mCherry (red). PmpC was immunolabeled using anti-PmpC antibodies (green). DNA was stained with DAPI (blue). White squares indicate regions of interest that are shown at higher magnification views (INSET), highlighting PmpC localization at the periphery of bacterial cells. Scale bars: 10 µm (mCherry/PmpC/DAPI) and 1 µm (INSET).

To further investigate PmpC localization, we employed super-resolution confocal microscopy. This approach allowed us to visualize PmpC’s distinctive peripheral localization around individual bacteria at all time-points examined (18, 24, 30, and 48 hpi). High-magnification inset images clearly showed that PmpC localizes to the bacterial surface, forming ring-like structures that outline the perimeter of mCherry-expressing *C. trachomatis* (Fig. 4).

Collectively, these findings identify PmpC as a surface-exposed protein that is expressed primarily throughout the mid-to-late stages of *C. trachomatis* development as well as under IFNγ- and penicillin-induced stress and recovery, while being partially released into the inclusion lumen.

### Lack of PmpC alters bacterial dynamics within the inclusion, delays host cell invasion, and impairs lateral spread

**Fig 5:**
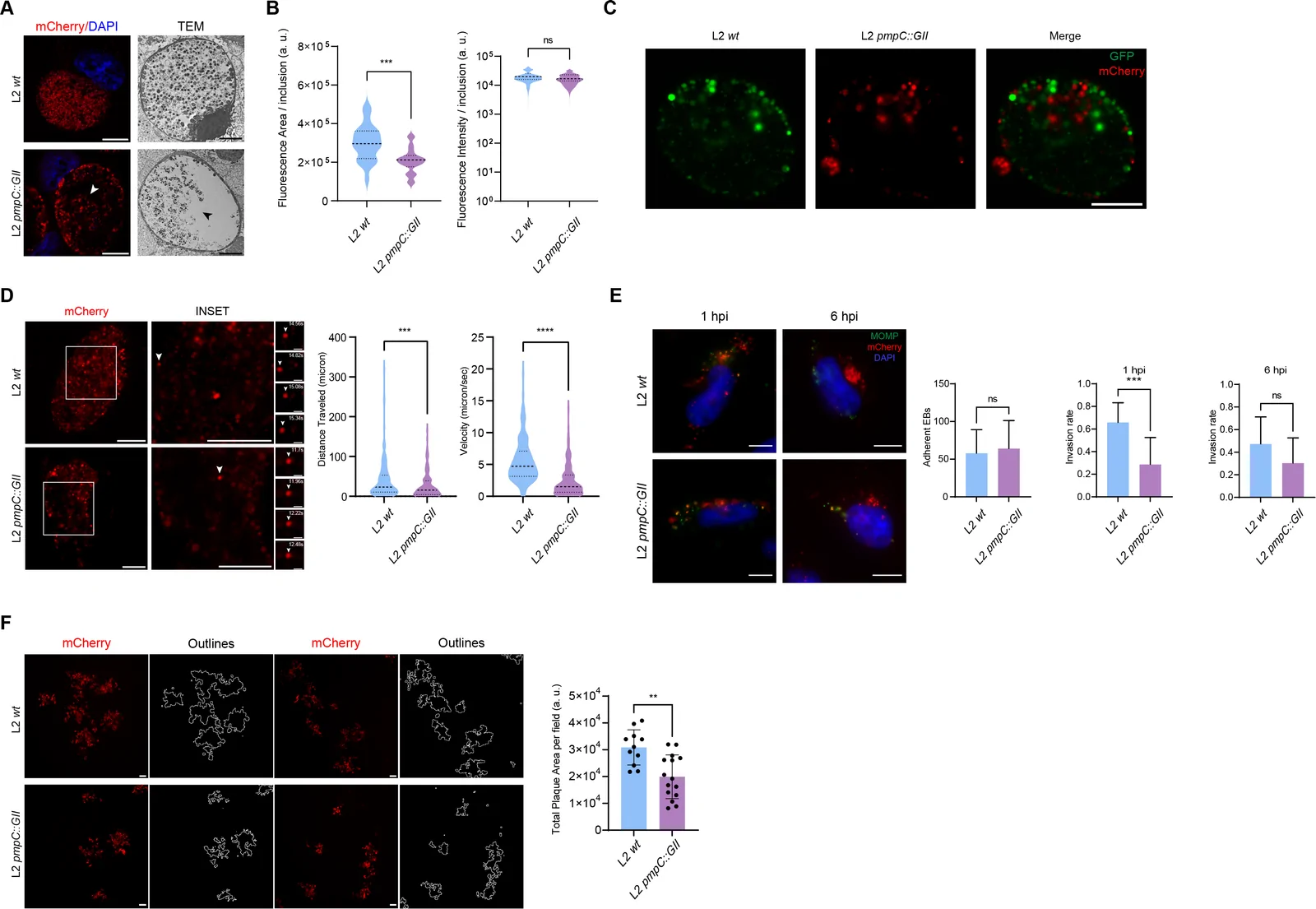
Lack of PmpC induces the formation of aggregates within the chlamydial inclusion, alters the dynamic behavior of bacterial cells within host cells, and delays invasion. (**A**) Representative confocal z-stack images (sum of slices projection) (left) and TEM images (right) of HeLa cells infected with *C. trachomatis* L2 *wt* and L2 *pmpC::GII* constitutively expressing mCherry (red) at 48 hpi. DNA was stained with DAPI (blue). Arrowheads indicate bacterial aggregates observed for L2 *pmpC::GII*. Scale bars: 10 μm. (**B**) Quantitative analysis of red fluorescence area and integrated fluorescence intensity per inclusion expressed as arbitrary units (a. U.), performed on images acquired using a Cytiva In-Cell Analyzer from infections carried out as in panel **A**. L2 *pmpC::GII* strain showed a 28% reduction in red fluorescence area compared with L2 *wt*. Image analysis was conducted using ImageJ software. Violin plots represent the median and interquartile range. Data were obtained from at least 20 inclusions. Statistical significance was calculated using Mann-Whitney *U* test (***P < 0.001, ns: not significant). (**C**) Representative images of super-resolution time-lapse video microscopy of inclusions co-infected with *C. trachomatis* L2 *wt* GFP (green) and L2 *pmpC::GII* mCherry (red) at 44 hpi, showing that L2 *pmpC::GII* aggregates are not rescued by the presence of L2 *wt* within the same inclusion. Scale bars: 10 μm. (**D**) Left: representative images of super-resolution time-lapse video microscopy of inclusions in HeLa cells infected with *C. trachomatis* L2 *wt* or L2 *pmpC::GII*, both constitutively expressing mCherry (red), at 44 hpi. White squares indicate regions of interest that are shown at higher magnification (INSET). Arrowheads highlight representative bacterial particles with their trajectories tracked over time (micrographs shown at higher magnification at the right, time expressed in seconds). Scale bars: 10 μm (mCherry and INSET), 1 μm (micrographs at the right). Right: quantitative analysis of the total distance traveled and average velocity of individual mCherry-positive bacteria (red) within inclusions. L2 *pmpC::GII* strain exhibited a 34% reduction in total distance traveled and a 68% reduction in average velocity compared with L2 *wt*. Violin plots represent the median and interquartile range. Data were obtained from at least 140 particles per condition. Asterisks indicate statistically significant differences as determined by Mann-Whitney *U* test (***P < 0.001, ****P < 0.0001). (**E**) Left: representative fluorescence images of HeLa cells infected with L2 *wt* or L2 *pmpC::GII* (MOI 10) constitutively expressing mCherry (red) at the indicated hpi. Extracellular bacteria adhered to non-permeabilized cells were labeled using anti-MOMP (green). DNA was stained with DAPI (blue). Scale bars, 10 μm. Right: quantitative analysis of adherent EBs (total red particles observed) per cell and invasion rates calculated (proportion of internalized bacteria) at the indicated time points. Bars represent the mean ± SD. Data were obtained from at least 20 cells. Statistical significance was assessed using the student’s *t*-test (***P < 0.001, ns: not significant). (**F**) Left: representative fluorescence images of plaque assays carried out on HeLa cells infected with L2 *wt* or L2 *pmpC::GII* strains (MOI 0.01), both constitutively expressing mCherry (red), and their corresponding outlines generated by ImageJ software (two images per condition are shown). Images were acquired at 20 days post-infection using a Cytiva InCell Analyzer 2500HS. Scale bars: 100 μm. Right: plaque area was quantified as total mCherry-positive area per field of view and expressed as arbitrary units (a. U.) using ImageJ software following threshold-based segmentation of the lysis regions. L2 *pmpC::GII* showed a 35% reduction in average plaque area. Bars represent the mean ± SD. Data points represent individual fields analyzed. Statistical significance was assessed using the Student’s *t*-test (**P < 0.01).

We next sought to determine the broader role of PmpC in *C. trachomatis* biology beyond stress recovery. First, we examined the spatial organization of bacteria within inclusions. Strikingly, we found that L2 *pmpC::GII* exhibited a distinctive clustered bacterial distribution characterized by uneven spatial organization and unpopulated luminal regions within the inclusion (Fig. 5A and B). Quantitative analysis supported these observations, showing a significant reduction in the total fluorescence area per inclusion compared with L2 *wt,* despite similar overall fluorescence intensities (Fig. 5B), along with a more than threefold increase in the frequency of inclusions displaying this altered distribution (Fig. S4). This phenotype suggested that PmpC might influence bacterial behavior through cell-to-cell interactions. To test whether the presence of PmpC-expressing bacteria could rescue this defect, we performed co-infections using L2 *wt* (expressing GFP) and L2 *pmpC::GII* (expressing mCherry). Live-cell microscopy revealed that even within the same inclusion, *pmpC*-null bacteria maintained their clustered phenotype despite the presence of PmpC-positive wild-type bacteria (Fig. 5C and Video S1). This suggests that the altered distribution of *pmpC* mutants within the inclusion cannot be complemented by neighboring wild-type bacteria. The abnormal clustering led us to investigate how the loss of PmpC affects bacterial dynamics within inclusions. Using time-lapse super-resolution microscopy, we tracked individual bacterial movements over time and quantified their motion parameters. Analysis of bacterial trajectories showed that L2 *pmpC::GII* exhibited a significant reduction in both total distance traveled and average velocity compared with L2 *wt* (Fig. 5D and Video S2). These results indicate that PmpC is necessary to maintain the characteristic distribution and movement of chlamydial particles within the inclusion lumen.

The surface localization of PmpC and its impact on bacterial dynamics led us to question whether this protein might influence early infection events, particularly bacterial adherence to and invasion of host cells, which are considered important virulence features. We quantified bacterial adhesion and invasion efficiency at early timepoints (1 and 6 hpi). While we found no significant difference in the number of adherent EBs per cell between the two strains, the invasion process was delayed in the *pmpC*-null mutant (Fig. 5E). At 1 hpi, the invasion rate of the mutant was significantly lower than that of the *wt*, though these levels tend to normalize by 6 hpi. This suggests that while PmpC is not essential for initial attachment, it enhances early invasion of host cells.

Finally, we integrated these observations—clustered distribution, reduced intra-inclusion movement, and delayed invasion—to assess their impact on the ability of the bacteria to disseminate through a host monolayer. We reasoned that the combination of these defects would likely impair lateral spread. Indeed, in plaque assays, the L2 *pmpC::GII* strain produced significantly smaller plaque area than L2 *wt* after 20 days of infection (Fig. 5F). Together, these findings demonstrate that while PmpC is dispensable for full adherence, its absence elicits a range of defects spanning bacterial spatial organization within the inclusion to delayed invasion and impaired spread to neighboring cells.

### PmpC is required for optimal bacterial replication and stress recovery in an *ex vivo* murine model of uterine infection

To assess the role of PmpC in a more physiologically relevant system, we used an *ex vivo* murine model of uterine infection in mouse uterine horn explants. This approach allowed us to examine PmpC function within complex tissue architecture while maintaining controlled experimental conditions.

**Fig 6:**
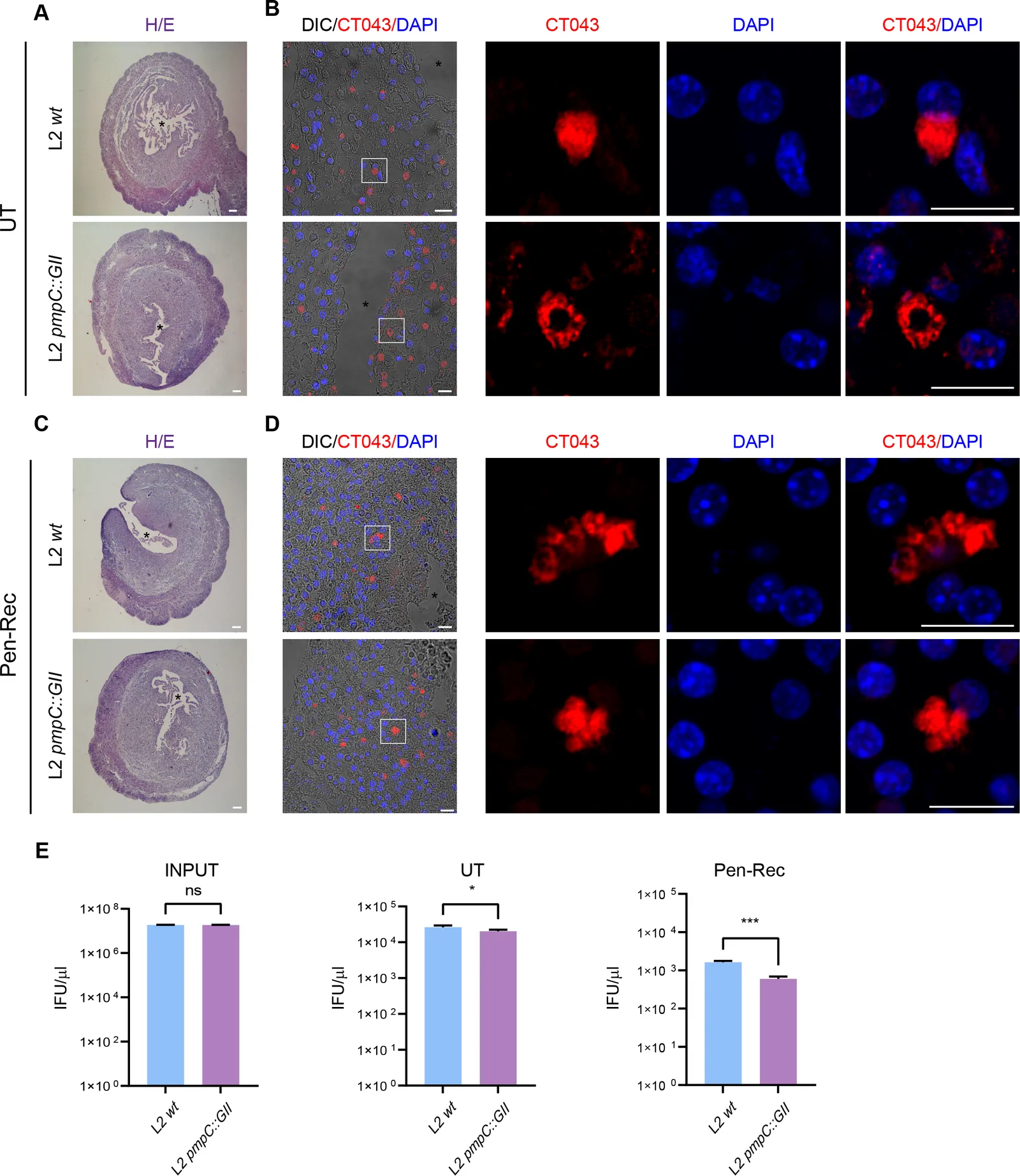
Lack of PmpC reduces progeny generation and recovery from penicillin-induced stress in an ex vivo murine model of uterine infection. (**A**) Representative histological images of uterine horn tissue sections from mice infected with L2 *wt* or L2 *pmpC::GII* (2 × 10⁷ EBs per sample) at 72 hpi stained with hematoxylin and eosin (H/E); UT: untreated. Scale bars, 100 μm. (**B**) Immunofluorescence images of the same tissues shown in panel **A**. Left: super-resolution fluorescence micrographs, showing differential interference contrast (DIC) images overlaid with bacterial labeling using anti-CT043 antibodies (red) and nuclear staining with DAPI (blue). White squares highlight regions of interest shown at higher magnification (right panels). Scale bars, 10 μm. (**C**) Representative histological images of uterine horn tissue sections from mice subjected to penicillin treatment followed by recovery (Pen–Rec) at 72 hpi, infected with L2 *wt* or L2 *pmpC::GII* stained with hematoxylin and eosin (H/E). Scale bars, 100 μm. (**D**) Immunofluorescence images of the same tissues shown in panel **C**. Left: super-resolution fluorescence micrographs, showing DIC images overlaid with bacterial labeling using anti-CT043 antibodies (red) and nuclear staining with DAPI (blue). White squares highlight regions of interest shown at higher magnification (right panels). Scale bars, 10 μm. (**E**) Quantification of infectious progeny generation, expressed as inclusion-forming units (IFUs). No significant differences (ns) were observed between L2 *wt* and L2 *pmpC::GII* in the input control (30 hpi, UT). L2 *pmpC::GII* exhibited a modest reduction (1.3-fold) in IFUs compared with L2 *wt* under UT conditions and a more pronounced reduction (2.7-fold) in IFU generation in the Pen–Rec condition in uterine horn explants. Data are presented as the mean ± SD from three replicates. Statistical significance was assessed using the Student’s *t*-test (*P < 0.05, ***P < 0.001, ns: not significant). (**A–D**) Asterisks highlight the uterine lumen.

We first established that both L2 *wt* and L2 *pmpC::GII* were capable of infecting the uterine tissue explants under untreated conditions (UT). Histological analysis using H/E staining revealed preserved tissue architecture with observable uterine lumens and no evidence of tissue degradation or gross structural distortion (Fig. 6A). The presence of chlamydial inclusions in the uterine horns at 72 hpi for both L2 *wt* and L2 *pmpC::GII* strains was confirmed by super-resolution immunofluorescence microscopy (Fig. 6B).

We next leveraged this model to examine the impact of PmpC during penicillin-induced stress. To this end, uterine explants were treated with penicillin at 24 hpi for a duration of 24 h, followed by a 24-h recovery period (Pen-Rec). H/E staining showed overall preservation of the architecture of the uterine tissue (Fig. 6C), and microscopic analysis corroborated that, during Pen-Rec, both strains developed inclusions containing organisms with the distinct morphological features of aberrant RBs (Fig. 6D), resembling what we observed in HeLa cell cultures (Fig. 2C).

Next, we quantified the impact of PmpC loss on the generation of infectious EBs. While no differences were observed between the strains in the input control (30 hpi), the L2 *pmpC::GII* mutant exhibited a modest but statistically significant reduction (1.3-fold decrease) in IFU generation compared with L2 *wt* in untreated explants (Fig. 6E). This finding suggests that in the context of primary uterine tissue, PmpC provides a subtle but measurable advantage. Remarkably, this defect was more evident in the Pen-Rec condition, where the L2 *pmpC::GII* mutant showed a more pronounced impairment in IFU production (2.7-fold reduction) compared with L2 *wt* (Fig. 6E). Altogether, these data underscore an important role for PmpC in facilitating the exit from penicillin-induced stress in the context of a complex female reproductive tract tissue environment.

## DISCUSSION

This study focused on the function of PmpC in *C. trachomatis*, a multifunctional virulence factor with roles extending beyond its previously reported function as an adhesin (60, 73, 88). We uncovered critical roles for this protein in stress recovery, bacterial intra-inclusion spatial organization, and dynamics, early invasion, and lateral spread.

In a genetic screen for mutants defective in IFNγ- and penicillin-induced stress recovery (74), we identified M111, a *C. trachomatis* strain carrying a nonsense mutation in *pmpC*, suggesting an unexpected role for PmpC in the stress recovery response. The ability of *C. trachomatis* to enter a reversible, non-replicating state—termed “chlamydial persistence” or “chlamydia stress response”—under immune pressure, nutritional deprivation, or antibiotics exposure has been linked to this bacterium’s ability to cause chronic infections (29, 89–91). Thus, our identification of PmpC as an important factor for recovery from IFNγ- and penicillin-induced stress reveals a new aspect of Pmps' function on chlamydial persistence and pathogenesis.

Previous studies have identified numerous chlamydial factors, including genes involved in tryptophan biosynthesis, the general stress response, RB to EB differentiation, cell envelope biogenesis, and cell division, that are differentially regulated during stress (41, 71, 92–97). However, genetic evidence linking these *C. trachomatis* stress response mechanisms to persistence remains scarce. Notable exceptions include the work by Kari et al., who utilized a tryptophan synthase subunit B (*trpB*)-null mutant to demonstrate its essential role in evading IFNγ-induced stress (98). Similarly, Muramatsu and colleagues conducted a large-scale screen linking missense mutations in *trpB*, the amino acid transporter *ctl0225*, and the hypothetical gene *ctl0194* to reduced capacity for recovery from IFNγ exposure (99). Furthermore, mutants defective in the putative protease Ptr/Ctl0175 indicated a link to impaired recovery from IFNγ- but not penicillin-induced stress (74).

Our findings indicate that the absence of PmpC impairs genome replication during the recovery phase, suggesting that this protein may influence the biological processes required to resume DNA synthesis following stress removal. Moreover, the failure to efficiently transition from RBs to EBs during recovery suggests that PmpC participates in the developmental program that coordinates a successful exit from the persistent state and the re-establishment of a productive infection.

PmpC is primarily expressed at mid to late stages of the developmental cycle and maintains expression during both the induction of and recovery from penicillin- and IFNγ-mediated stress. These findings are consistent with two previous proteomics studies of RBs and EBs (23) and RBs, EBs, and IFNγ-induced aRBs (71). Similarly, a transcriptomic profiling study during standard growth and IFNγ-induced stress and reactivation detected *pmpC* constant mRNA at all analyzed time points (12, 24, and 48 hpi) (100), and previous work on the transcriptional regulation of *pmp* genes found that *pmpC* mRNA is predominantly expressed during mid-to-late development (18-48 hpi) (87). The observation that PmpC expression occurs predominantly at stages when bacteria transition from RB to EB under standard growth, and is maintained throughout stress induction and recovery phases, together with the defective stress recovery of the *pmpC*-null mutant, positions PmpC as an important player in chlamydial reactivation.

Based on super-resolution microscopy analysis, PmpC localizes to the bacterial surface, forming distinctive ring-like structures around individual bacteria. This finding is consistent with the known membrane association of Pmp autotransporter family members (59, 60, 62, 67, 69, 87, 88). Imaging and fractionation experiments indicated that PmpC is also released into the inclusion lumen during infection. Interestingly, a similar finding was previously reported for PmpD (67), which has also been detected in the soluble fraction of infected cells, consistent with the secretion of a subset of Pmp proteins outside bacterial cells. The decreasing colocalization of PmpC with bacterial cells observed at later time points suggests a progressive release of PmpC. The presence of PmpC in the extrabacterial space could serve several potential functions: it might act as an effector molecule coordinating bacterial behavior within inclusions or facilitate modification of the inclusion environment to support bacterial survival and replication. It could also gain access to the extracellular environment upon lysis of infected cells and interact with host proteins, as has been suggested for PmpD (69).

One of our most intriguing findings was the *pmpC*-null phenotype characterized by a clustered distribution within inclusions. This observation, along with impaired intraluminal bacterial motion properties, provides evidence that PmpC expression is required for proper bacterial spatial organization within the inclusion lumen. Interestingly, Kari and coworkers (101) characterized a *C. trachomatis pmpD*-null mutant and similarly observed an atypical intra-inclusion distribution. Their description of areas lacking chlamydial organisms within the inclusion closely resembles the clustered phenotype we identified for the *pmpC*-null strain. The finding that the loss of PmpC or PmpD results in altered spatial distribution suggests that maintaining bacterial organization within the inclusion may be a conserved function shared by multiple members of the Pmp family. *Chlamydia* are non-motile and lack flagella; however, they have been shown to undergo movement within the inclusion (102), most likely due to Brownian motion. Consequently, the reduced motion observed may be a direct physical consequence of bacterial clustering, which restricts the passive displacement of individual organisms within the inclusion lumen.

The observation that co-infection experiments between *pmpC*-null mutant and wild-type strains failed to restore bacterial dispersal within the inclusion suggests that surface-associated PmpC, rather than the released fraction, drives this altered spatial distribution. While the molecular mechanisms underlying PmpC’s role in this process remain to be fully elucidated, these results suggest that PmpC’s interactions with itself or other cell-surface proteins prevent bacterial clustering. Regardless of the mechanism, we reasoned that this phenotype could impair the efficiency of lateral spread to neighboring cells by physically hindering lytic exit, thereby diminishing the capacity to initiate secondary infections. This is consistent with the observation that the *pmpC*-null mutants form smaller plaque areas in cell monolayers, even though they do not display replication defects in the absence of stress. The impaired lateral spread observed *in vitro* may have implications for the efficiency of bacterial dissemination within host tissues; however, this remains to be experimentally determined.

The delayed invasion phenotype observed in the *pmpC*-null mutant indicates that this protein influences early infection events. The temporal pattern of this defect, characterized by reduced efficiency at one hpi, suggests that PmpC facilitates the kinetics of invasion rather than being required for entry. While the specific contribution of PmpC to *C. trachomatis* host-cell invasion has not been characterized previously, a role for PmpD in both adherence and invasion has been proposed (101, 103). Similarly, studies on *C. pneumoniae* Pmp21, a homolog of *C. trachomatis* PmpD, have proposed a role for this protein in bacterial attachment and host-cell entry, likely mediated by interactions with the EGF receptor (104). All nine *C. trachomatis* Pmps have been implicated as adhesins (60, 88, 101) and likely partially compensate for the absence of PmpC during bacterial attachment. This functional redundancy suggests that while PmpC is dispensable for adherence, it fulfills a more specialized role in facilitating the kinetics of invasion. However, as invasion normalizes by six hpi and baseline infectious progeny production is unaltered, the smaller plaque phenotype most likely supports a role for PmpC in preventing bacterial clustering that hinders lateral spread.

*Ex vivo* tissue models offer advantages for pathogenesis studies by serving as a physiological bridge between simplified cell cultures and whole-animal systems. They preserve the complex three-dimensional architecture and multicellular diversity of the host environment (features absent in 2D cultures) while providing a more cost-effective, ethically manageable, and experimentally tractable alternative to animal models. Several studies have successfully used *ex vivo* human fallopian tube models to investigate *C. trachomatis* pathogenesis (105–109). Given that mice are widely accepted animal models for investigating the pathogenesis of *C. trachomatis* genital infections (110, 111), an *ex vivo* murine uterine horn model represents a valuable physiologically relevant platform. Our validation of key findings in cell culture using this *ex vivo* model represents, to our knowledge, its first application to investigate *C. trachomatis* infection under both standard growth conditions and antibiotic-induced stress recovery. We chose penicillin as the stress inducer rather than IFNγ for several practical and biological reasons. First, IFNγ-mediated immune responses to *C. trachomatis* differ between humans and mice (112, 113), complicating the standardization and interpretation of results in murine systems. Second, an effective *ex vivo* model for stress recovery must allow for precise, controlled induction and reversal of stress. Currently, there is no practical method to reliably “turn on and off” IFNγ-induced stress within a complex tissue explant. In contrast, penicillin treatment provides a tractable system to manipulate chlamydial stress and has been successfully exploited in prior *in vivo* studies examining amoxicillin-induced persistence in mice (55). The observation that under standard growth conditions the *pmpC*-null mutant exhibits a significant reduction in infectious progeny generation, in contrast to cell culture, where no growth defect was observed, suggests that the complex tissue environment unmasks functional requirements for PmpC that are not captured in simplified culture systems. The amplified *pmpC*-null phenotype observed during recovery from penicillin-induced stress confirms PmpC’s role in facilitating exit from persistent states and further suggests that PmpC may play tissue-specific functions in the female genital tract during *C. trachomatis* infection.

A limitation of this study is that the *pmpC*-null mutant could not be genetically complemented. Repeated attempts at plasmid-based complementation were unsuccessful, which is consistent with known technical constraints associated with expressing a 5,325-bp gene encoding a large surface-exposed protein in an organism with limited genetic tractability such as *C. trachomatis*. Similar obstacles have been reported by other investigators when attempting to complement PmpD expression in a *pmpB*-null mutant (101). In addition, although TEM remains one of the most reliable approaches for direct ultrastructural identification of chlamydial developmental forms, it is subject to inherent limitations. These include sampling bias associated with two-dimensional sectioning of heterogeneous inclusions, as well as reliance on morphological criteria. Accordingly, TEM-based quantitative assessments should be interpreted with caution and, when possible, considered alongside independent complementary approaches, such as reporter-based fluorescence methods or functional readouts provided by IFU assays.

### Conclusions

This study defines *C. trachomatis* PmpC as a multifunctional virulence factor with important roles in (i) recovery from IFNγ- and penicillin-induced stress, (ii) maintaining proper bacterial spatial distribution within inclusions, (iii) facilitating the kinetics of early invasion processes, and (iv) supporting bacterial lateral spread to adjacent cells. In addition, its surface localization and progressive release into the inclusion lumen position PmpC as a potential mediator of bacterial-environment interactions. Finally, the findings presented in this work significantly expand our understanding of PmpC function and provide new insights into the complex mechanisms underlying chlamydial pathogenesis, persistence, and virulence.

## Data Availability

All data that support the results of this study are available upon request via the corresponding author.

## SUPPLEMENTAL MATERIAL

Fig. S1 spectrum.01773-26-s0001.pdf A genetic screen identifies *C. trachomatis* mutants impaired for recovery from IFNγ- and penicillin-induced stress. Mutants of *C. trachomatis* exhibiting reduced infectious progeny expressed as infectious forming units normalized per input (IFU/Input), were observed upon recovery from stress induced by IFNγ (IFN-Rec) or penicillin (PEN-Rec) (gray and red dots, *n* = 8) compared with the wild-type *C. trachomatis* LGV-L2 (L2 *wt*, black dot). Among these, the M111 mutant (red dot) consistently displayed reduced IFUs during recovery from IFNγ- and PEN-induced stress and was selected for further analysis.

Fig. S2 spectrum.01773-26-s0002.pdf PCR confirmation of insertional disruption of *pmpC*. (A) Schematic representation of *pmpC* and *aadA-GII* intron used for pmpC disruption, as indicated. PCR primers used for molecular analysis of *pmpC* disruption (black arrows) and predicted insertion site are shown. bp: base pairs. (B) PCR reactions were performed on genomic DNA from L2 *wt* and L2 *pmpC::GII* strains to assess disruption of *pmpC*. Gel 1: expected amplified band of 1200 bp for *aadA* (aadA_F and aadA_R primers) for intron detection regardless of orientation, is observed in L2 *pmpC::GII* only. Gel 2: expected amplified bands of 367 bp for L2 *wt* and 2,579 bp if insertion occurred for L2 *pmpC::GII* (FcheckTargpmpC and RcheckTargpmpC primers) are observed. Gel 3: as control, *ompA* was amplified and the expected 103 bp PCR product was detected in both strains. M: DNA marker. bp: base pairs.

Fig. S3 spectrum.01773-26-s0003.pdf Transcript-level analysis of *pmpABC* operon in L2 *wt* and L2 *pmpC::GII*. (A) Schematic representation of *pmpABC* operon, primers used for RT-PCR reactions, and predicted insertion site, as indicated. (B) Endpoint RT-PCR was performed on cDNA from L2 *wt* and L2 *pmpC::GII* strains to assess transcription of *pmpA*, *pmpB*, *pmpC*, and *aadA* genes. Gel 1: the expected amplified bands for both L2 *wt* and L2 *pmpC::GII* are 590 bp for *pmpA* (pmpA gene_F and pmpA gene_R primers); 598 bp for *pmpB* (pmpB gene_F and pmpB gene_R primers); and 555 bp for *pmpC* (pmpC gene_F, pmpC gene_R primers). PC: genomic DNA from L2 *wt* used as positive control. NTC: no template control. Gel 2: 1,200 bp for intron (aadAF and aada_R primers), regardless of orientation, observed only in L2 *pmpC::GII*. Gel 3: expected bands of 367 bp for L2 *wt* and 2,579 bp for L2 *pmpC::GII* (FcheckTargpmpC and RcheckTargpmpC primers) are observed. M: DNA marker. bp: base pairs.

Fig. S4 spectrum.01773-26-s0004.pdf Lack of PmpC leads to clustered bacterial distribution within the chlamydial inclusion. (A) Representative fluorescence images of HeLa cells infected with *C. trachomatis* L2 *wt* and L2 *pmpC::GII* constitutively expressing mCherry (red). Images were acquired at 40 hpi using a Cytiva InCell Analyzer 2500HS (40×). Host and chlamydial DNA were stained with DAPI (blue). White arrows highlight L2 *pmpC::GII* inclusions displaying an altered phenotype characterized by bacterial clustering and empty luminal spaces. Scale bars: 10 μm. (B) Percentage of inclusions displaying bacterial clustering from the conditions shown in panel A. At least 100 inclusions per condition were analyzed. Asterisks indicate a statistically significant difference (*****P* < 0.0001, Chi-square test).

Table S1 spectrum.01773-26-s0005.docx Single nucleotide variants present in the genome of M111.

Table S2 spectrum.01773-26-s0006.docx Primers used in this study.

Video S1 spectrum.01773-26-s0007.avi L2 *pmpC::GII* aggregates are not rescued by the presence of L2 wt within the same inclusion. Super resolution time-lapse video microscopy corresponding to the representative images shown in Fig. 5C, displaying inclusion in HeLa cell co-infected with *C. trachomatis* L2 *wt* GFP (green) and L2 *pmpC::GII* mCherry (red) at 44 hpi. The movie was generated from 200 images acquired every 0.26 s (52 s total).

Video S2 spectrum.01773-26-s0008.avi Lack of PmpC alters the dynamic behavior of bacterial cells within the inclusion. Super resolution time-lapse video microscopy corresponding to the quantitative analysis shown in Fig. 5D, depicting individual mCherry-positive *C. trachomatis* L2 *wt* or L2 *pmpC::GII* particles expressing mCherry (red) within the inclusion in HeLa cells. The movie was generated from 200 images acquired every 0.26 s (52 s total).

ASM does not own the copyrights to Supplemental Material that may be linked to, or accessed through, an article. The authors have granted ASM a non-exclusive, world-wide license to publish the Supplemental Material files. Please contact the corresponding author directly for reuse.
