# Supplementary material for: Loss of PmpC in Chlamydia trachomatis leads to altered bacterial development and impaired recovery from IFNγ- and penicillin-induced stress
Source: Microbiol Spectr. 2026 Oct 12;14(11):e01773-26. doi: 10.1128/spectrum.01773-26 (PMC13729156; doi:10.1128/spectrum.01773-26)
Supplement: Supplementary file 1 [file spectrum.01773-26-s0001.pdf]

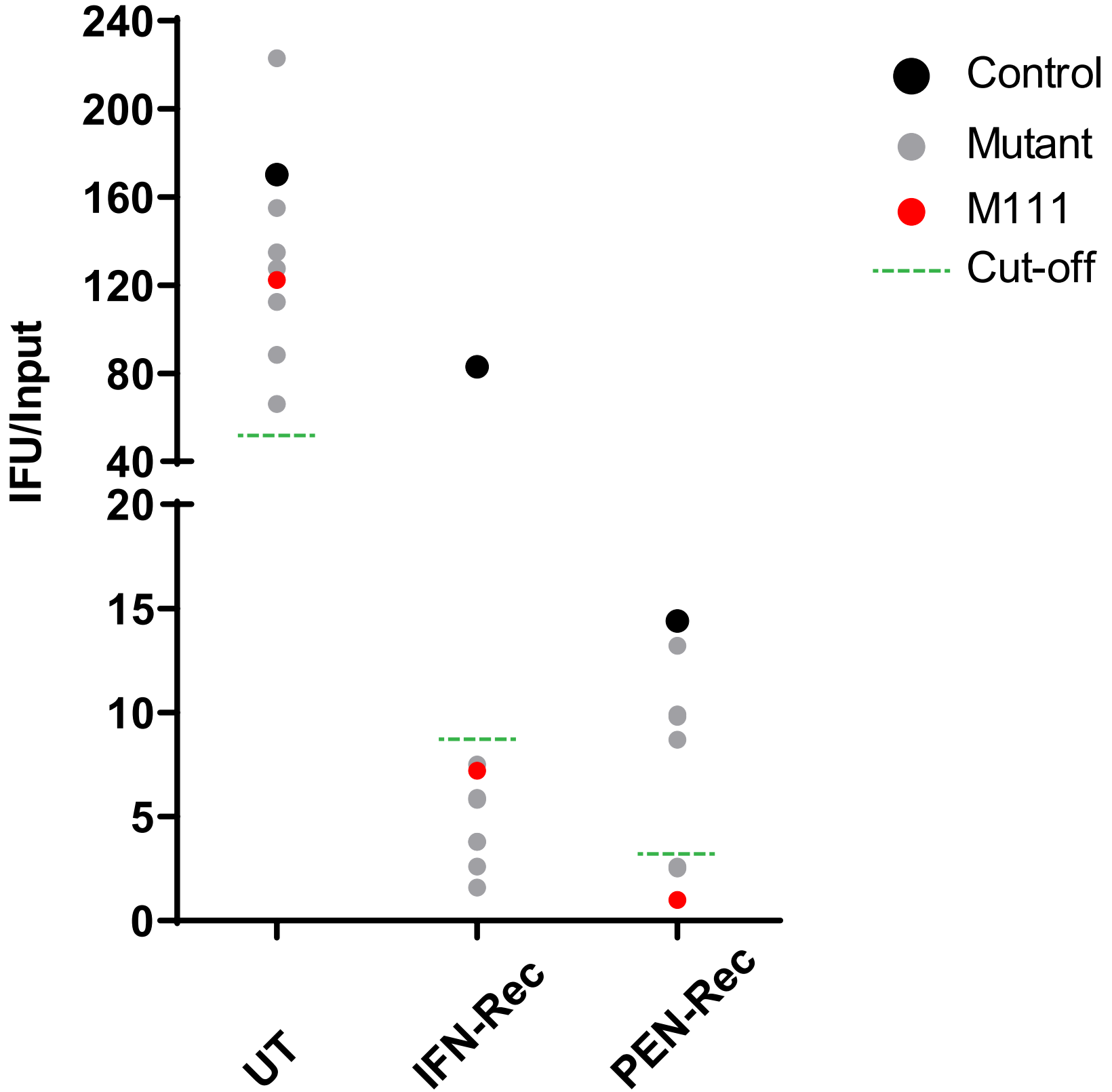

240
200
160
120
80
40
20
15
10
5
0
IFU/Input
UT
IFN-Rec
PEN-Rec
Control
Mutant
M111
Cut-off
