# Supplementary material for: Loss of PmpC in Chlamydia trachomatis leads to altered bacterial development and impaired recovery from IFNγ- and penicillin-induced stress
Source: Microbiol Spectr. 2026 Oct 12;14(11):e01773-26. doi: 10.1128/spectrum.01773-26 (PMC13729156; doi:10.1128/spectrum.01773-26)
Supplement: Supplementary file 2 [file spectrum.01773-26-s0002.pdf]

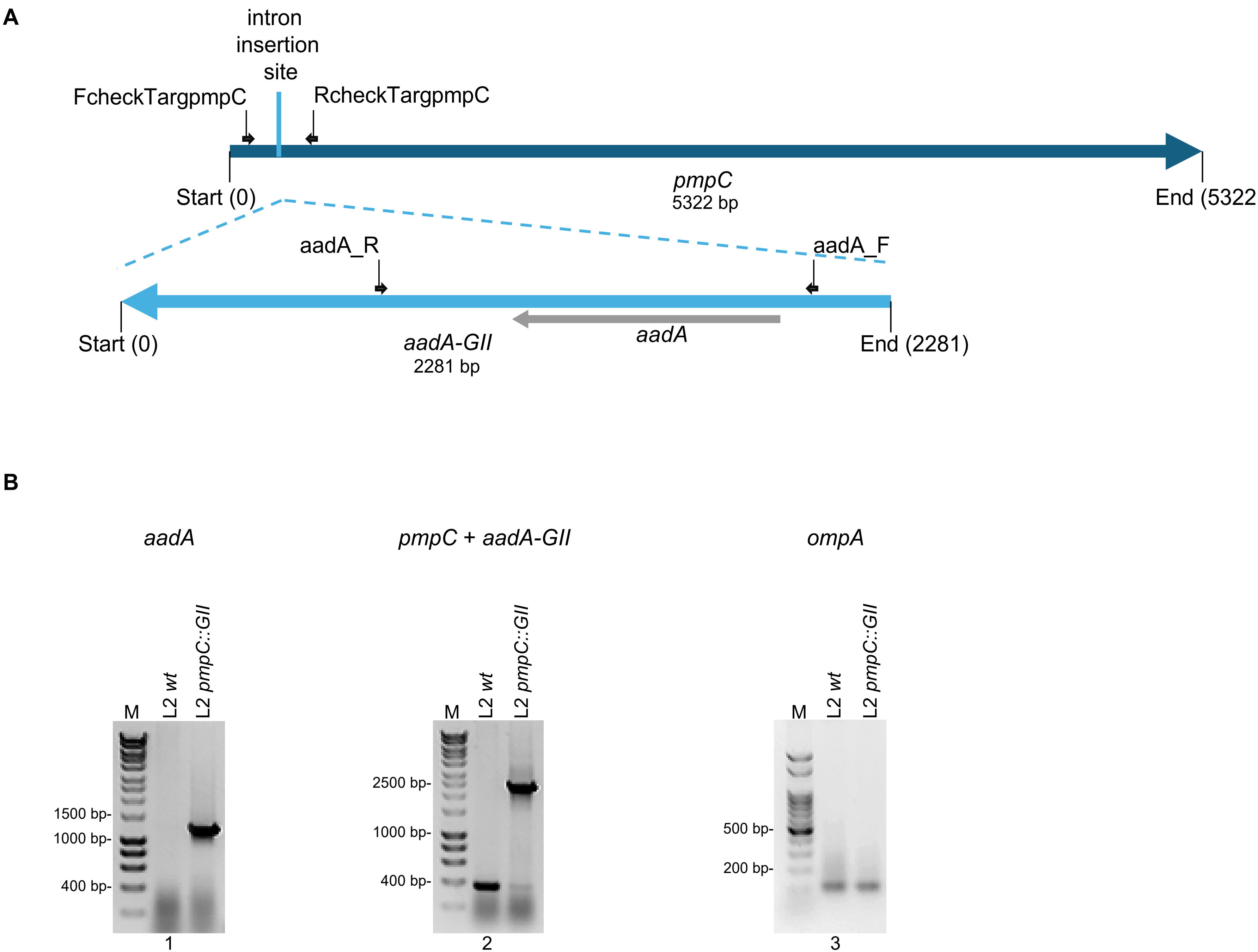
A
intron insertion site
FcheckTargpmpC
RcheckTargpmpC
Start (0)
pmpC
5322 bp
End (5322)
aadA_R
aadA_F
Start (0)
aadA-GII
2281 bp
aadA
End (2281)
B
aadA
pmpC + aadA-GII
ompA
M
L2 wt
L2 pmpC::GII
1500 bp-
1000 bp-
400 bp-
2500 bp-
1000 bp-
400 bp-
500 bp-
200 bp-
1
2
3
