# Supplementary material for: Loss of PmpC in Chlamydia trachomatis leads to altered bacterial development and impaired recovery from IFNγ- and penicillin-induced stress
Source: Microbiol Spectr. 2026 Oct 12;14(11):e01773-26. doi: 10.1128/spectrum.01773-26 (PMC13729156; doi:10.1128/spectrum.01773-26)
Supplement: Supplementary file 3 [file spectrum.01773-26-s0003.pdf]

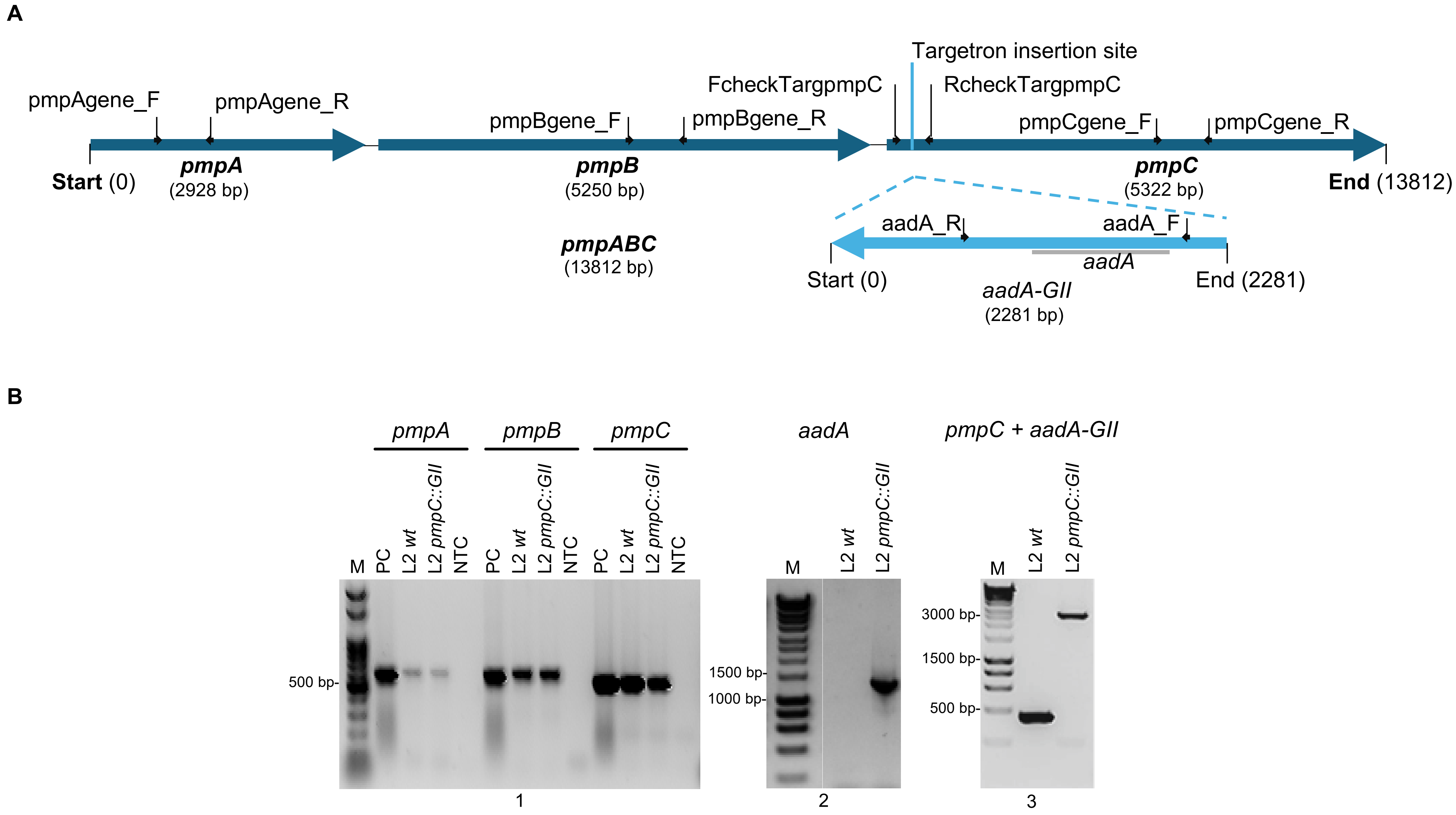
A
Targetron insertion site
FcheckTargpmpC
RcheckTargpmpC
pmpAgene_F
pmpAgene_R
pmpBgene_F
pmpBgene_R
pmpCgene_F
pmpCgene_R
Start (0)
pmpA
(2928 bp)
pmpB
(5250 bp)
pmpC
(5322 bp)
End (13812)
pmpABC
(13812 bp)
aadA_R
aadA_F
aadA
Start (0)
End (2281)
aadA-GII
(2281 bp)
B
pmpA
pmpB
pmpC
aadA
pmpC + aadA-GII
M
PC
L2 wt
L2 pmpC::GII
NTC
500 bp-
1500 bp-
1000 bp-
3000 bp-
1
2
3
