# Supplementary material for: Loss of PmpC in Chlamydia trachomatis leads to altered bacterial development and impaired recovery from IFNγ- and penicillin-induced stress
Source: Microbiol Spectr. 2026 Oct 12;14(11):e01773-26. doi: 10.1128/spectrum.01773-26 (PMC13729156; doi:10.1128/spectrum.01773-26)
Supplement: Supplementary file 4 [file spectrum.01773-26-s0004.pdf]

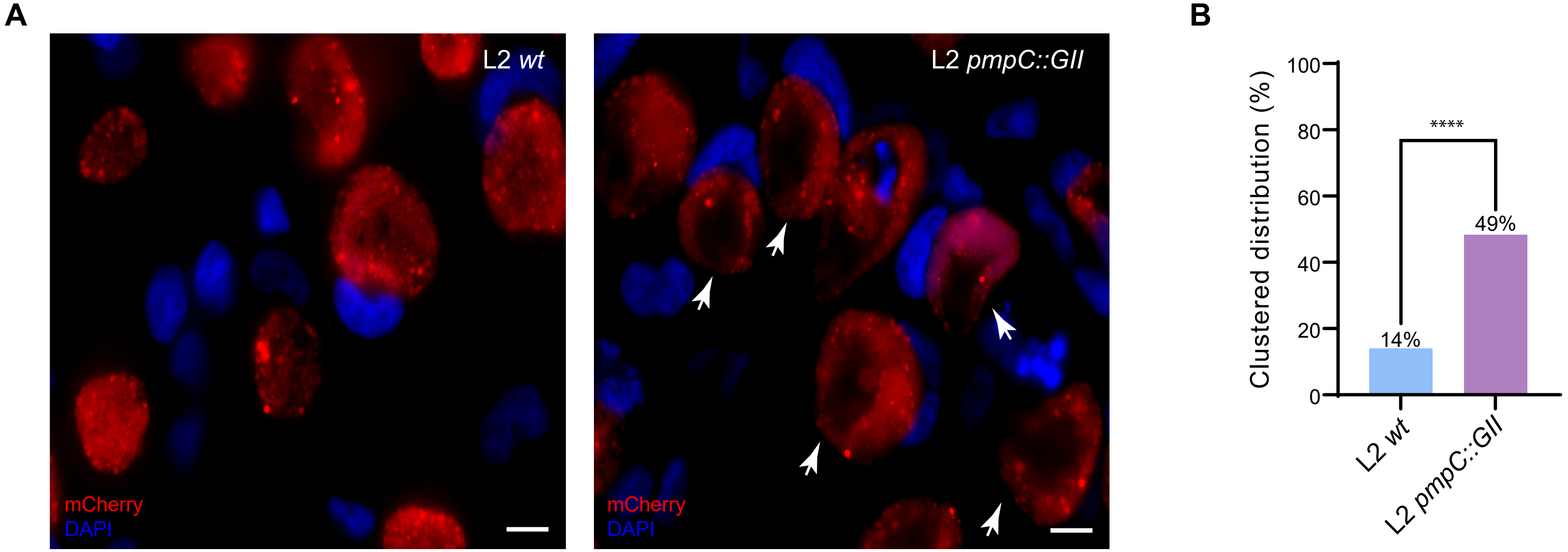

A
L2 *wt*
mCherry
DAPI
L2 *pmpC::GII*
mCherry
DAPI
B
Clustered distribution (%)
100
80
60
40
20
0
****
14%
49%
L2 *wt*
L2 *pmpC::GII*
