# Supplementary material for: Loss of PmpC in Chlamydia trachomatis leads to altered bacterial development and impaired recovery from IFNγ- and penicillin-induced stress
Source: Microbiol Spectr. 2026 Oct 12;14(11):e01773-26. doi: 10.1128/spectrum.01773-26 (PMC13729156; doi:10.1128/spectrum.01773-26)
Supplement: Supplementary file 5 [file spectrum.01773-26-s0005.docx]

| **Table S1. Single nucleotide variants present in the genome of M111.** | | | | |
| --- | --- | --- | --- | --- |
| **Genome  position^1^** | **Base pair substitution^2^** | **Gene** | **Locus ID^3^** | **Amino acid substitution** |
| 123.163 | C > A |  |  |  |
| 127.338 | T657A |  | CTL0103 |  |
| 435.453 | G743A | *nusA* | CTL0352 | A248V |
| 448.523 | G110A |  | CTL0364 | A37V |
| 616.827 | C315T |  | CTL0518 |  |
| 637.039 | C610T | *clpC* | CTL0538 | R204C |
| 638.353 | T1924C | *clpC* | CTL0538 | F642L |
| 675.519 | G1411A | *rpoB* | CTL0567 | H471Y |
| 693.265 | G473A | *pykF* | CTL0586 | S158N |
| 798.820 | G470A | *pmpC* | CTL0671 | G157E |
| 929.717 | C > A |  |  |  |
| 59.991 | C > T |  |  |  |
| 94.181 | G93A | *tig* | CTL0076 |  |
| 119.658 | G155A |  | CTL0096 | A52V |
| 208.194 | G>A |  |  |  |
| 364.375 | G120A |  | CTL0291 |  |
| 414.928 | G190A | *folD* | CTL0334 | P64S |
| 419.583 | G289A |  | CTL0339 | P97S |
| 537.830 | C > T |  |  |  |
| 801.411 | C3061T | *pmpC* | CTL0671 | N1020* |
| Mutations shaded in gray are present in the parental RifR LGV-L2 434/Bu strain compared to the original LGV-L2 434/Bu reference genome, as previously described (1). ^1^Genome position of base pair substitution induced by EMS mutagenesis (Kokes et al., 2015). ^2^Base pair substitution in coding domain sequence. *^3^C. trachomatis* LGV-L2 434/Bu reference genome locus ID. Red colour highlights the mutation causing a premature stop codon in *pmpC.* | | | | |

**References**

1.	Nguyen BD, Valdivia RH. 2012. Virulence determinants in the obligate intracellular pathogen *Chlamydia trachomatis* revealed by forward genetic approaches. Proc Natl Acad Sci U S A 109:1263-8.
