# Supplementary material for: Loss of PmpC in Chlamydia trachomatis leads to altered bacterial development and impaired recovery from IFNγ- and penicillin-induced stress
Source: Microbiol Spectr. 2026 Oct 12;14(11):e01773-26. doi: 10.1128/spectrum.01773-26 (PMC13729156; doi:10.1128/spectrum.01773-26)
Supplement: Supplementary file 6 [file spectrum.01773-26-s0006.docx]

**Table S2. Primers used in this study.**

| Primer | Sequence (5’ to 3’) |
| --- | --- |
| PmpC_1_IBS 1/2 | AAAAAAGCTTATAATTATCCTTAGAAGACACTCCTGTGCGCCCAGATAG GGTG |
| PmpC_1_EB S1/delta | CAGATTGTACAAATGTGGTGATAACAGATAAGTCACTCCTTCTAACTTA CCTTTCTTTGT |
| PmpC_1_EB S2 | TGAACGCAAGTTTCTAATTTCGATTTCTTCTCGATAGAGGAAAGTGTCT |
| Universal | CGAAATTAGAAACTTGCGTTCAGTAAAC |
| MOMP_F | TTTGCCGCTTTGAGTTCTGC |
| MOMP_R | CGCCGAAACCTTCCCATAGA |
| aadA_F | GTAACGCGTCCCGGGCCTGATAGTTTGGCTGTGAG |
| aadA_R | TCTACGCGTTGCCTGACGATGCGTGGAG |
| FcheckTargpmpC | ACCGACTGCAATGTTAGCAA |
| RcheckTargpmpC | TGTCATGGAGTTCTATGCTTGGA |
| pmpAgene_F | ACTTCGCAGGATCCAGAATTCT |
| pmpAgene_R | TTTGTTCGCTGCAAGTTGGA |
| pmpBgene_F | AGATCTTCCGATGCTGGAAC |
| pmpBgene_R | TTTGTCCAGATGTTGCAGAA |
| pmpCgene_F | ATCAGGAGGGGCTCATTTCT |
| pmpCgene_R | GTTTTCCCTTTTGCAGCTTG |
| FHisp-PmpC2 | GCGCCATGGATGGTGGGGTTATTCATTCTAAAACGG |
| RHisp-PmpC2 | CGAGGCCTTTATGGCACCAACCCAATAGCAGA |
